# High-Fat Diet Changes Fungal Microbiomes and Interkingdom Relationships in the Murine Gut

**DOI:** 10.1128/mSphere.00351-17

**Published:** 2017-10-11

**Authors:** Timothy Heisel, Emmanuel Montassier, Abigail Johnson, Gabriel Al-Ghalith, Yi-Wei Lin, Li-Na Wei, Dan Knights, Cheryl A. Gale

**Affiliations:** a Department of Pediatrics, University of Minnesota, Minneapolis, Minnesota, USA; b Université de Nantes, EA 3826 Thérapeutiques cliniques et expérimentales des infections, Nantes, France; c The BioTechnology Institute, University of Minnesota, St. Paul, Minnesota, USA; d Bioinformatics and Computational Biology, University of Minnesota, Minneapolis, Minnesota, USA; e Department of Pharmacology, University of Minnesota, Minneapolis, Minnesota, USA; f Department of Computer Science and Engineering, University of Minnesota, Minneapolis, Minnesota, USA; Arizona State University

**Keywords:** fungal-bacterial interactions, fungi, high-fat diet, microbiome, obesity

## Abstract

Dietary fat intake and shifts in gut bacterial community composition are associated with the development of obesity. To date, characterization of microbiota in lean versus obese subjects has been dominated by studies of gut bacteria. Fungi, recently shown to affect gut inflammation, have received little study for their role in obesity. We sought to determine the effects of high-fat diet on fungal and bacterial community structures in a mouse model using the internal transcribed spacer region 2 (ITS2) of fungal ribosomal DNA (rDNA) and the 16S rRNA genes of bacteria. Mice fed a high-fat diet had significantly different abundances of 19 bacterial and 6 fungal taxa than did mice fed standard chow, with high-fat diet causing similar magnitudes of change in overall fungal and bacterial microbiome structures. We observed strong and complex diet-specific coabundance relationships between intra- and interkingdom microbial pairs and dramatic reductions in the number of coabundance correlations in mice fed a high-fat diet compared to those fed standard chow. Furthermore, predicted microbiome functional modules related to metabolism were significantly less abundant in high-fat-diet-fed than in standard-chow-fed mice. These results suggest a role for fungi and interkingdom interactions in the association between gut microbiomes and obesity.

**IMPORTANCE** Recent research shows that gut microbes are involved in the development of obesity, a growing health problem in developed countries that is linked to increased risk for cardiovascular disease. However, studies showing links between microbes and metabolism have been limited to the analysis of bacteria and have ignored the potential contribution of fungi in metabolic health. This study provides evidence that ingestion of a high-fat diet is associated with changes to the fungal (and bacterial) microbiome in a mouse model. In addition, we find that interkingdom structural and functional relationships exist between fungi and bacteria within the gut and that these are perturbed by high-fat diet.

## Abstract (precis)

Recent research shows that gut microbes are involved in the development of obesity, a growing health problem in developed countries that is linked to increased risk for cardiovascular disease. However, studies showing links between microbes and metabolism have been limited to the analysis of bacteria and have ignored the potential contribution of fungi in metabolic health. This study provides evidence that ingestion of a high-fat diet is associated with changes to the fungal (and bacterial) microbiome in a mouse model. In addition, we find that interkingdom structural and functional relationships exist between fungi and bacteria within the gut and that these are perturbed by high-fat diet.

## INTRODUCTION

Emerging evidence demonstrates that diet, both independently and in conjunction with body composition, modulates gut microbial community structure (1, 2). The resulting microbiome modulations from high-fat diet in particular influence the risk for development of obesity and obesity-related disorders such as diabetes and atherosclerosis (3–5). Proposed mechanisms for microbially mediated obesity effects include increased energy extraction from the diet (6, 7), production of neuroactive factors that decrease satiety or increase hunger (8, 9), and induced inflammatory responses (10).

The majority of studies examining the links between gut microbes and metabolic health have focused almost exclusively on the bacterial component of the microbiota (11–13), even though microbes from other microbial kingdoms are present within the intestinal tract. In particular, diverse fungal communities also colonize the intestinal tract of humans and mice (14–19). Importantly, perturbation of bacterial communities, for example by antibacterial antibiotics, changes fungal communities as well as bacterial communities (14), underlining the importance of considering interkingdom interactions in studies seeking to understand microbiome-mediated effects on human health.

Fungi have been reported to contribute less than 1% of the microbes in the intestinal tract of humans (20, 21); however, this number likely underestimates fungal numbers and importance. Of note, there is underrepresentation of fungal sequences in annotated reference sequence databases. As of July 2017, the National Center for Biotechnology Information (NCBI) database (22) listed 1,069 complete fungal genomes compared to 14,249 complete bacterial genomes, and so fungi may be underdetected by shotgun sequencing efforts. In addition, fungal cells are more than 100-fold larger than typical bacterial cells; thus, they provide a substantial mass of biomaterial and unique metabolic functions to the microbiota as well as surface area for host-microbe interactions. Although they represent a small component of the microbiota, fungal communities are significant in that they potentially serve as a reservoir for pathogens as well as for keystone species with critical roles in maintaining the function of the gut microbiome (23). Furthermore, so-called minor components of the microbiota can nevertheless proliferate under certain host conditions, such as those occurring with antibiotic exposure, with significant health consequences for the host.

In this study, we sought to gain an understanding of how an obesogenic diet affects host-associated microbes, beyond that which has been described for only one microbial kingdom, the bacteria. We hypothesized that fungal microbiomes and, specifically, fungal-bacterial microbiome interactions involved in metabolism would be disrupted by high-fat diet. Overall, the results of this study provide evidence that fungi and interkingdom interactions are disrupted by high-fat diet, thus supporting the inclusion of fungal community analyses in studies that seek to discover new connections between intestinal microbiomes and metabolic health.

## RESULTS

### Mice fed a high-fat diet harbor gut fungal and bacterial communities that differ from those in mice fed standard chow.

Mice fed a high-fat diet had increased weight gain and metabolic markers associated with obesity, such as insulin resistance, compared to mice fed standard chow (see Table S1 at http://galelab.umn.edu/msphere-supplemental-material), which is consistent with the findings of previous studies utilizing a mouse model of diet-induced obesity (24). High-fat diet in this study consisted of 60% calories from fat, and regular chow contained 18% calories from fat.

**FIG 1:**
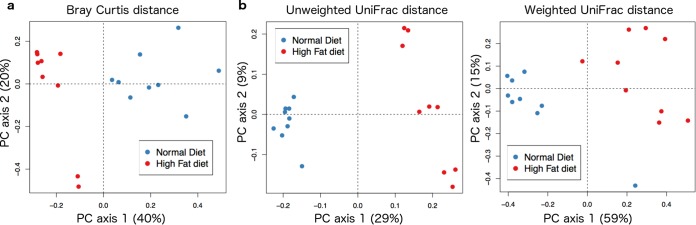
Beta-diversity comparisons of fungal communities (a) and bacterial communities (b) of feces from mice fed high-fat and standard diets. PCoA of Bray-Curtis distances is shown for fungi, and weighted (right) and unweighted (left) UniFrac distances are shown for bacteria. The proportion of variance explained by each principal coordinate is denoted in the corresponding axis label.

Mice fed high-fat and standard diets have comparable levels of identified fungal taxonomies in their feces as indicated by alpha-diversity metrics for Chao1 (*t* test using Monte Carlo permutations, *P* = 0.384), Shannon’s index (*t* test, *P* = 0.79), and number of observed unique species (*t* test using Monte Carlo permutations, *P* = 0.439). However, principal-component analysis (PCoA) showed separation of microbial communities by diet, as measured using Bray-Curtis distance metrics (analysis of similarities [ANOSIM], *R* = 0.864, *P* = 0.001) (Fig. 1a). Furthermore, using randomForest (25), unknown samples were classified with a 0.056 ± 0.17 error rate, which is nine times better than the baseline error rate for random guessing, showing that the fecal fungal microbiotas are distinct between the two diets.

High-fat diet was associated with decreased bacterial richness and diversity, as measured by phylogenetic diversity–whole-tree (*t* test using Monte Carlo permutations, *P* = 0.04) and Shannon (*t* test using Monte Carlo permutations, *P* = 0.04) indices (see Fig. S1 in the supplemental material). Moreover, PCoA showed separation by diet, as measured using unweighted UniFrac distance metrics (ANOSIM, *R* = 0.99, *P* = 0.01) and weighted UniFrac distance metrics (ANOSIM, *R* = 0.75, *P* = 0.01) (Fig. 1b). Furthermore, using randomForest, unknown samples were classified with an 0.056 ± 0.017 error rate, which is nine times better than the baseline error rate for random guessing, showing that fecal bacterial microbiotas are distinct between the two diets.

10.1128/mSphere.00351-17.1 FIG S1  Bacterial alpha-diversities (phylogenetic distance–whole-tree method [a] and Shannon diversity index method [b]) were plotted based on rarefaction of the number of bacterial samples. Download FIG S1, PDF file, 0.6 MB. Copyright © 2017 Heisel et al. 2017 Heisel et al. This content is distributed under the terms of the Creative Commons Attribution 4.0 International license.

**FIG 2:**
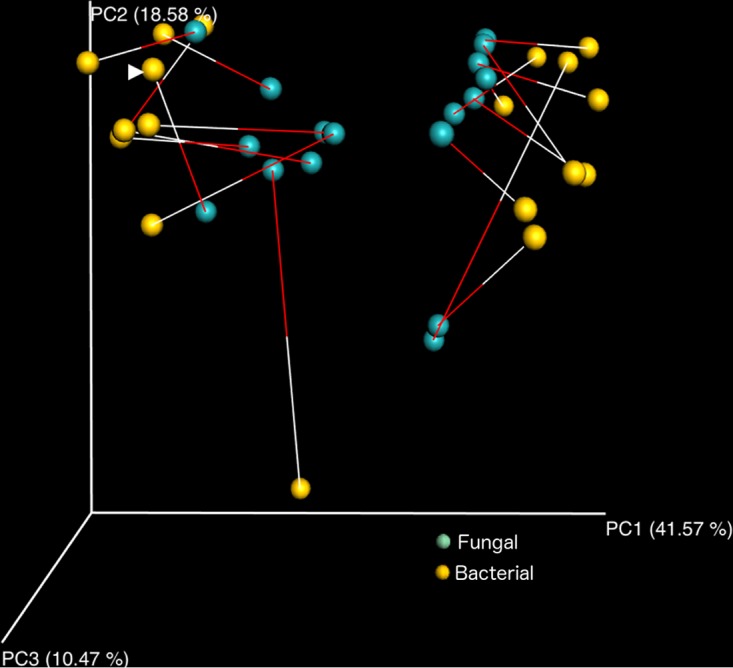
Procrustes analysis comparing the spatial fit of unweighted UniFrac principal-coordinate matrices of bacterial communities (yellow spheres) and Bray-Curtis principal-coordinate matrices of fungal communities (green spheres) from mice fed high-fat and standard diets. Concordance was observed between bacterial and fungal profile changes in response to diet (*M*^2^ = 0.513, *P* < 0.01).

**FIG 3:**
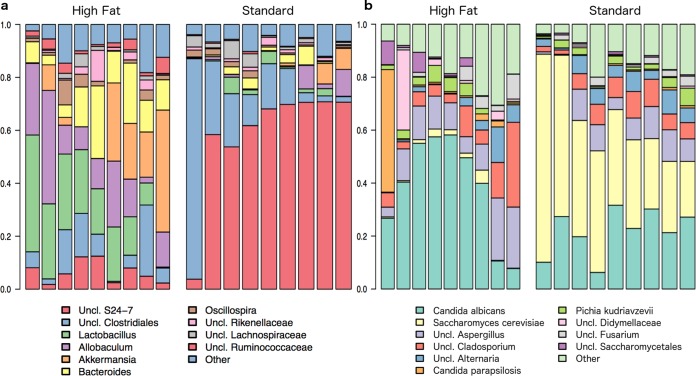
Abundance plots of sequencing results. (a) Relative abundance plots of bacterial taxa in mouse feces. Taxa were identified to 97% similarity using the Greengenes reference database, as described in Materials and Methods. (b) Relative abundance plots of fungal taxa in mouse feces. Taxa were identified to the species level using the UNITE reference database, as described in Materials and Methods. For the results of statistical comparisons of taxon abundances between diets, see Fig. S2 and S4 in the supplemental material.

Procrustes analysis showed concordance between PCoA plots from the unweighted UniFrac distance metrics (bacteria) and the Bray-Curtis distance metrics (fungal), indicating consistency between bacterial and fungal profiles (*M*^2^ = 0.623, *P* < 0.001) (Fig. 2). In comparing diet groups, the plots of relative abundances of bacteria at genus level (Fig. 3a) and fungi at species level (Fig. 3b) were both visually distinct.

We observed that the relative abundances of 19 bacterial (genus-level) and 6 fungal (genus- or species-level) taxa were significantly different (Mann-Whitney U test, false discovery rate [FDR]-corrected *P* value of <0.05) between the two diets. For bacteria, mice fed a high-fat diet had increased *Bacteroides*, *Enterococcus*, *Proteus*, *Lactobacillus*, *Ruminococcaceae*, *Streptococcus*, *Christensenellaceae*, and *Allobaculum* numbers and decreased *Prevotella*, *Anaeroplasma*, *Erysipelotrichaceae*, *Turicibacter*, and “*Candidatus* Arthromitus” numbers (Fig. S2) compared to mice fed standard chow. Overall, these taxon changes represent an increase in *Firmicutes* and a decrease in *Bacteroidetes* in response to high-fat diet (Fig. S3) and are consistent with the results of other investigators (7, 26).

10.1128/mSphere.00351-17.2 FIG S2  Bee swarm analyses were performed on all bacterial taxa, comparing abundances for each taxon between diets and testing for significant differences. The plots shown are from comparisons that showed significant differences (FDR-corrected *P* value of <0.05). Download FIG S2, PDF file, 0.1 MB. Copyright © 2017 Heisel et al. 2017 Heisel et al. This content is distributed under the terms of the Creative Commons Attribution 4.0 International license.

10.1128/mSphere.00351-17.3 FIG S3  Relative abundance plot of bacterial phyla, showing a decrease in *Bacteroidetes* and an increase in *Firmicutes* in mice fed a high-fat diet compared to a standard diet. Download FIG S3, PDF file, 0.01 MB. Copyright © 2017 Heisel et al. 2017 Heisel et al. This content is distributed under the terms of the Creative Commons Attribution 4.0 International license.

For fungi, the abundances of the *Alternaria*, *Saccharomyces*, *Septoriella*, and *Tilletiopsis* genera; *Saccharomyces cerevisiae*; and *Tilletiopsis washingtonensis* were higher in mice fed standard chow (Fig. S4; FDR-corrected *P* value of <0.05), while no taxa were significantly higher in mice fed the high-fat chow. The numbers of differentiated taxa increased with less-stringent FDR-corrected *P* value cutoffs (Fig. S5; FDR-corrected *P* value of <0.25), with six taxa showing higher relative abundances in the high-fat-diet mice and 20 taxa showing higher abundances in the standard-chow mice.

10.1128/mSphere.00351-17.4 FIG S4  Bee swarm analyses were performed on all fungal taxa, comparing abundances for each taxon between diets and testing for significant differences. The plots shown are from comparisons that showed significant differences (FDR-corrected *P* value of <0.05). Download FIG S4, PDF file, 0.03 MB. Copyright © 2017 Heisel et al. 2017 Heisel et al. This content is distributed under the terms of the Creative Commons Attribution 4.0 International license.

10.1128/mSphere.00351-17.5 FIG S5  Bee swarm analyses were performed on all fungal taxa, comparing abundances for each taxon between diets and testing for significant differences using a more permissive FDR-corrected *P* value. Plots shown here are from those comparisons that showed significant differences (FDR-corrected *P* value of <0.25). Download FIG S5, PDF file, 0.05 MB. Copyright © 2017 Heisel et al. 2017 Heisel et al. This content is distributed under the terms of the Creative Commons Attribution 4.0 International license.

*Candida albicans*, the predominant colonizer and fungal pathogen of humans, was detected in both groups of mice (average relative abundances of 38.5% and 22.0% for high-fat-diet- and standard chow-fed mice, respectively; not statistically different, FDR-corrected *P* value of 0.30). This is in conflict with previous reports stating that *C. albicans* is not a commensal of the intestinal tract of mice (reviewed in reference 27). To confirm this finding in our samples, we performed quantitative real-time PCR (qPCR) analysis using *C. albicans* species-specific primers on a subset of the samples from mice fed the standard chow. qPCR confirmed the presence of small amounts of *C. albicans* DNA in all samples (see Table S2 at http://galelab.umn.edu/msphere-supplemental-material) and not in the negative control, providing evidence that *C. albicans* sequences are present in the feces of mice analyzed in this study.

### Fungal microbiomes of mouse feces differ from those present in the corresponding mouse chow.

**FIG 4:**
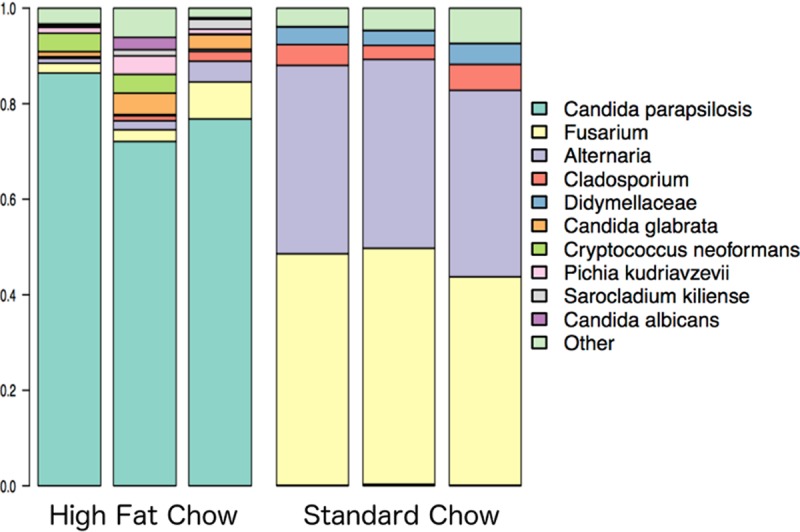
Relative abundance plots of fungal taxa for mouse chows. Each chow was sequenced three individual times (DNA was isolated from different pieces of chow for each sequencing run), and results for each chow were pooled after sequencing. Fungi were identified to the species level, as described in Materials and Methods.

To determine the extent to which the mouse chows used in this study contributed to mouse fungal microbiomes, fungal DNA was extracted from the high-fat and standard chows and analyzed using qPCR and sequencing. By qPCR analysis, the standard chow contained a much larger amount of fungal DNA than did the high-fat chow (mean quantification cycle [*C*_*q*_] values of 22 and 36, respectively). Overall, standard chow contained a larger number of fungal taxa than did high-fat chow (231 and 69 fungal taxa, respectively). In comparing fungal microbiomes of mice and their corresponding chows, we observed that ~80% of fungal taxa in mice fed the standard diet were not present in the standard chow and ~90% of fungal taxa in mice fed the high-fat diet were not present in the high-fat chow. The most abundant taxa in standard chow were the *Fusarium* (46% of sequences) and *Alternaria* (39% of sequences) genera (Fig. 4). In contrast, in mice fed the standard chow, *Fusarium* and *Alternaria* contributed 2% and 5%, respectively, of fungal sequences. Similarly, the most abundant taxon in mice fed standard chow was *S. cerevisiae*, which accounted for >40% of the sequences; this taxon accounted for <0.01% of sequences in the standard chow. In the high-fat chow, the most abundant taxon was identified as *Candida parapsilosis*, which accounted for 81% of all sequences, compared to only 6% of sequences in mice fed this diet. The most abundant taxon in mice fed the high-fat diet was *C. albicans*, which accounted for 38% of fungal sequences; this taxon accounted for only 1% of sequences in the high-fat chow. qPCR analysis of DNA isolated from the high-fat chow confirmed a small amount of *C. albicans* DNA. Altogether, these results provide evidence that mouse fecal fungal microbiomes are not solely structured by diet.

### Diet-specific intrakingdom coabundance correlations exist in the mouse gut.

Positive and negative coabundance correlations were observed between fungal taxa in the feces of both high-fat- and standard chow-fed mice (see Tables S3 and S4 at http://galelab.umn.edu/msphere-supplemental-material; also Fig. S6 and S7). In mice fed standard chow, there were a total of 57 significant fungal-fungal correlations (FDR-corrected *P* value of <0.25); 6 correlations were negative, and the remaining 51 were positive. In contrast, the number of coabundance correlations was reduced in mice fed a high-fat diet, where there were only 14 significant correlations; one correlation was negative, and the remaining 13 were positive. No correlations were shared between diets.

10.1128/mSphere.00351-17.6 FIG S6  Heat maps depicting bacterial-bacterial (a) and fungal-fungal (b) coabundance relationships in mice fed standard chow and bacterial-bacterial (c) and fungal-fungal (d) coabundance relationships in mice fed a high-fat diet with the color gradient (scale inset) indicating the strength and direction of the correlation. Red, positive correlation; blue, negative correlation. Download FIG S6, PDF file, 0.3 MB. Copyright © 2017 Heisel et al. 2017 Heisel et al. This content is distributed under the terms of the Creative Commons Attribution 4.0 International license.

10.1128/mSphere.00351-17.7 FIG S7  Network maps of fungal-fungal (a) and bacterial-bacterial (b) interactions in mice fed standard chow and fungal-fungal (c) and bacterial-bacterial (d) interactions in mice fed a high-fat diet. These maps visually show positive (blue line) and negative (gray line) correlations between either fungi or bacteria. Blue line, positive correlation; gray line, negative correlation. Nodes are positioned using an edge-weighted spring-embedded layout. For the fungal-fungal interaction network: red node, *Ascomycota* phylum; green node, *Basidiomycota* phylum. For the bacterial-bacterial interaction network: purple node, *Firmicutes* phylum; green node, *Bacteroidetes* phylum; yellow node, *Proteobacteria* phylum; gray node, *Verrucomicrobia* phylum; pink node, *Deferribacteres* phylum; dark blue node, *Cyanobacteria* phylum; light blue node, *Actinobacteria* phylum. Download FIG S7, PDF file, 2.7 MB. Copyright © 2017 Heisel et al. 2017 Heisel et al. This content is distributed under the terms of the Creative Commons Attribution 4.0 International license.

Positive and negative coabundance correlations were also observed between bacterial taxa in both the high-fat- and standard chow-fed mice (see Tables S5 and S6 at http://galelab.umn.edu/msphere-supplemental-material; also Fig. S6 and S7). In total, there were 23 negative and 39 positive correlations in the standard-chow-fed mice. Similarly to the fungal-fungal network analysis, the number of bacterial-bacterial coabundance correlations was decreased in mice fed a high-fat diet, with only 10 positive and no negative correlations observed.

### Diet-specific fungal-bacterial interkingdom coabundance correlations exist in the mouse gut.

**FIG 5:**
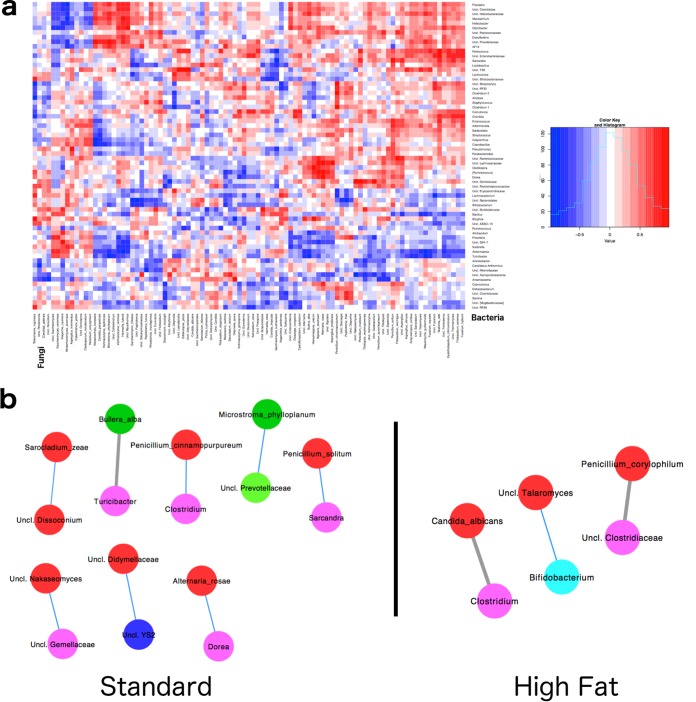
Interkingdom interactions between fungi and bacteria. (a) Heat map depicting fungal-bacterial coabundance relationships in mice fed standard chow with the color gradient (scale inset) indicating the strength and direction of the correlation. Red, positive correlation; blue, negative correlation. (b) Network maps of fungal-bacterial interactions in mice fed either standard or high-fat chow. Blue line, positive correlation; gray line, negative correlation. Nodes are positioned using an edge-weighted spring-embedded layout. Color coding is as follows: fungal nodes, red node, *Ascomycota* phylum; green node, *Basidiomycota* phylum; bacterial nodes, purple node, *Firmicutes* phylum; green node, *Bacteroidetes* phylum; pink node, *Deferribacteres* phylum; dark blue node, *Cyanobacteria* phylum; light blue node, *Actinobacteria* phylum.

Positive and negative interkingdom coabundance relationships were also observed between fungal and bacterial taxa in the gut, for both standard- and high-fat-diet-fed mice (Fig. 5). In mice fed standard chow, there were 8 significant fungal-bacterial coabundance correlations: 7 were positive, and 1 was negative (Fig. 5; see also Table S7 at http://galelab.umn.edu/msphere-supplemental-material). In mice fed the high-fat diet, there was a reduction in coabundance correlations, with only 3 being observed (one positive and two negative) (Fig. 5b; see also Table S8 at http://galelab.umn.edu/msphere-supplemental-material). The specific interkingdom taxon correlations completely differed between the diets. Altogether, these data support the idea that interkingdom interactions exist between bacteria and fungi in the gut and that these relationships depend upon diet.

### Fungal taxa correlate with bacterial KEGG modules.

**FIG 6:**
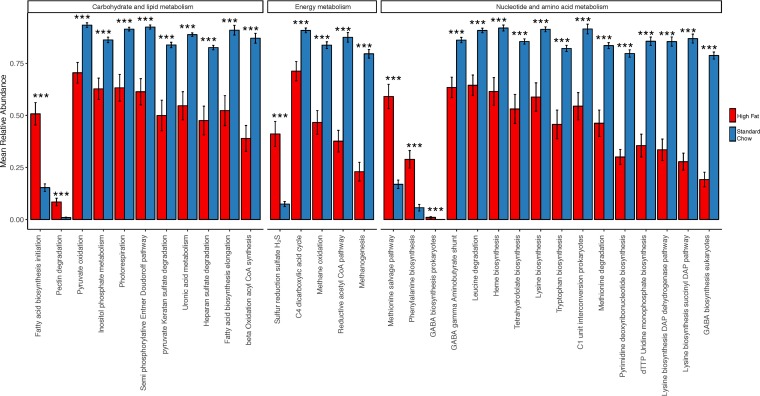
Functional microbiome differences between high-fat and standard chow diets. Relative abundance of each KEGG module was predicted with BugBase. KEGG modules displayed are metabolic modules that differ between high-fat and standard chow diets. KEGG modules are shown grouped by KEGG category. Asterisks indicate significant differences (Mann-Whitney U test; ***, FDR-corrected *P* value of <0.001; mean ± standard error).

**FIG 7:**
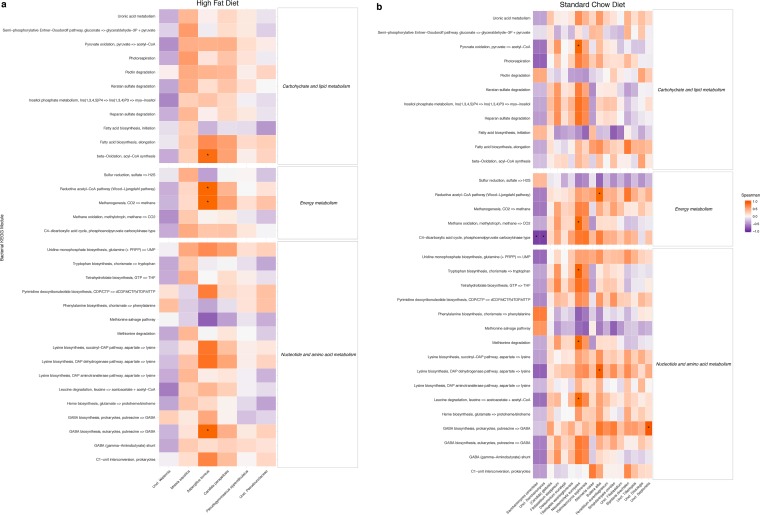
Correlations between bacterial KEGG modules and fungal taxa that increase with high-fat (a) and standard chow (b) diet. KEGG modules were limited to those that were significantly different between diet groups for testing. Taxa were limited to those that significantly increased with each dietary treatment as described in Materials and Methods. Positive correlations are depicted in red, and negative correlations are in blue. Asterisks indicate significant correlations (Spearman correlation; *, FDR-corrected *P* value of <0.05).

Inference of fungal microbiome function from sequence data is not technically possible due to the paucity of fungal sequences in current databases, complexities of fungal taxonomy calling, and variability in functions expressed by fungi depending on specific environmental conditions, compared to bacterial sequences. Nevertheless, we were able to use BugBase (28) to infer bacterial community functions of mouse gut microbiomes in order to identify potential links between fungi and metabolic potential of gut bacterial microbiomes. Of a total of 558 potential Kyoto Encyclopedia of Genes and Genomes (KEGG) functional modules (29), we determined that the relative abundances of 46 modules were significantly different between the high-fat- and standard-diet-fed mice (Mann-Whitney U test, FDR-corrected *P* value of <0.001). Of these, we subsequently focused our downstream analysis on those related to energy metabolism; of these, the majority of functional modules were decreased in mice fed the high-fat diet compared to standard chow (Fig. 6). To determine if any fungal taxa were correlated with bacterial function, we compared the relative abundances of significantly increased fungal taxa with KEGG modules within each diet group. In the high-fat-diet mice, we identified significant positive correlations between *Aspergillus terreus* and bacterial gamma-aminobutyric acid (GABA) biosynthesis, methanogenesis, the reductive acetyl coenzyme A (acetyl-CoA) pathway, and beta-oxidation (Fig. 7a; Spearman correlation, FDR-corrected *P* value of <0.05). In the standard-chow mice, *Saccharomyces* taxa were negatively correlated with the C_4_-dicarboxylic acid cycle; *Neoascochyta europaea* was positively correlated with leucine and methionine degradation, tryptophan biosynthesis, methane oxidation, and pyruvate oxidation; *Bullera alba* was positively correlated with lysine biosynthesis and the reactive acetyl-CoA pathway; and *Septoriella* was positively correlated with GABA biosynthesis (Fig. 7b; Spearman correlation, FDR-corrected *P* value of <0.05). We also predicted microbial community phenotypes using BugBase (e.g., “forms biofilms” and “stress tolerant”) from mouse microbiomes and found that 8 of the 9 phenotypes were significantly different between diets (Fig. S8). We also tested for correlations between the fungal taxa that were significantly different between diets and BugBase phenotypes to detect relationships that persist across the two dietary treatments and identified one potential positive correlation between the *Dioszegia* taxon and the anaerobic bacterial phenotype (permutation test of Spearman correlation test statistics, FDR-corrected *P* value of 0.2). While each dietary group had different correlation patterns, there were no correlations between differentiated fungal taxa and BugBase phenotypes within each diet that reached statistical significance (permutation test, Fig. S9).

10.1128/mSphere.00351-17.8 FIG S8  BugBase predictions for microbiome phenotypes and their corresponding OTU contributions at the phylum level by diet. Plots are included for all predicted phenotypes. Significance determined by Mann-Whitney U test is indicated on each plot. Download FIG S8, PDF file, 0.3 MB. Copyright © 2017 Heisel et al. 2017 Heisel et al. This content is distributed under the terms of the Creative Commons Attribution 4.0 International license.

10.1128/mSphere.00351-17.9 FIG S9  Heat map of correlations between BugBase-predicted phenotypes and fungal taxa. Maps are ordered by hierarchical clustering of rows by their similarity and columns by their similarity. Download FIG S9, PDF file, 0.01 MB. Copyright © 2017 Heisel et al. 2017 Heisel et al. This content is distributed under the terms of the Creative Commons Attribution 4.0 International license.

## DISCUSSION

In this study, ingestion of a high-fat diet by mice resulted in major shifts in both gut bacterial and gut fungal community structures. Although this has been shown previously for bacterial microbiomes (7, 26), this is the first report to our knowledge showing that fungal microbiomes are also affected by high-fat diet. In humans, fungal microbiomes have been reported to be influenced by diet (30), but not high-fat diet specifically, and to differ between obese and lean adults and children (18, 31). In addition, modulation of gut fungal community structure has been reported in association with intestinal inflammation (32–35), a process that potentially contributes to the development of obesity and the metabolic syndrome. These results, along with the results that we report here, support the idea that gut fungal communities could contribute to metabolic health.

The high-fat diet provided to the mice in this study contained 60% calories from fat compared to 18% for standard chow. This high level of fat in a human diet would be extreme and encountered rarely but is used in rodent models to induce obesity because it allows the animals to gain more weight at a higher rate (36, 37), thus allowing researchers to screen for effects after a shorter period of time. Additional studies are needed to understand the effects on the mycobiome of diets containing ranges of fat content, as well as differences in fat types (e.g., saturated versus unsaturated), that are more typically consumed by human populations.

In lean mice fed a standard chow, we observed that the fungal species *S. cerevisiae* was significantly more abundant than in obese mice fed a high-fat diet. This is consistent with a recent report showing that *Saccharomyces* species had significantly lower abundance in the gut of obese children than in controls (31). It remains unclear, however, whether the *S. cerevisiae* abundance difference has a direct role in the expression of obesity-associated phenotypes by the host. In support of a mechanistic connection between gut fungi and metabolic health, the administration of either chitin or β-glucan (polysaccharide components of the cell walls of many fungal species, including *S. cerevisiae*) or live *Saccharomyces boulardii* (an asporogenous form of *S. cerevisiae* [38]) to mice was shown to prevent obesity phenotypes induced by high-fat diet (39–41). These effects were associated with modulation of gut bacterial community structure; fungal microbiomes were not analyzed. Other fungal species have also been implicated in beneficial effects on the host. For example, metabolites from the *Penicillium* genus have been reported to exhibit anti-inflammatory and insulin-sensitizing activities (42). Altogether, these results support the hypothesis that gut fungal communities interact with the host to affect metabolism, possibly via fungus-derived molecules and/or by modulation of bacterial community functions.

Our analysis of chow microbiomes revealed that they differ from the fungal microbiomes of the murine gut. We observed that, although some fungal species were shared between gut and chow, many gut fungi were not present in the chow. These results indicate that recently ingested food is not the only source for gut fungi. Other potential sources include other chows ingested by the mice prior to purchase, animal bedding, and maternal sites with transfer of fungi occurring during birth and early life. Additionally, the differences in relative abundances between fungi in chow and the same fungal taxa found in the gut show that modulation of ingested fungal communities occurs within the mammalian gut. Open questions remain as to how fungal communities are acquired and mature over time and which fungi persist in the mammalian gut over the long term.

In this study, intra- and interkingdom coabundance relationships existed in the murine gut and these relationships were specific to diet type. Our functional analysis using BugBase and KEGG pathways provides further evidence of shared physiochemical interactions between bacteria and fungi and points to potential functional relationships that may be disrupted by dietary changes. In most environments, fungi and bacteria coexist and interact physically, communicate with each other via molecules and induction of signaling pathways, modulate their shared physiochemical environment, and compete with or complement each other with respect to nutrients, metabolism, and phoresis (43). Because of these interkingdom interactions, mixed microbial communities often have functional properties that are significantly different from each single-kingdom community (44, 45). For example, in human subjects ingesting a high-carbohydrate diet, a syntrophic relationship was observed between the fungal genus *Candida* and bacterial genera *Prevotella*, *Ruminococcus*, and *Methanobrevibacter* (30). The investigators proposed that digestion of dietary starch by *Candida* results in the release of simple sugars, which are fermented by *Prevotella* and *Ruminococcus*. *Methanobrevibacter* then completes the breakdown of fermentation by-products to carbon dioxide and/or methane. In our study, mice fed a high-fat diet had increased relative abundance of the fungus *Aspergillus terreus*, a fungus that produces the cholesterol-reducing agent lovastatin (46), and this correlated with increases in bacterial genes involved in carbon energy metabolism (methanogenesis, acetyl-CoA synthesis, and reductive acetyl-CoA pathways), implying a compensatory response to exposure to high-fat diet. In a recent study, rats receiving *A. terreus* as a dietary supplement along with a high-fat diet had reduced hepatic steatosis compared to those fed a high-fat diet alone (47), further supporting a role for *A. terreus* in modulation of metabolism. There is also evidence for fungal-bacterial relationships in other scenarios that affect health, for example, administration of *S. boulardii* to treat antibiotic-associated diarrhea caused by the bacterium *Clostridium difficile* (48) as well as intestinal inflammation caused by the bacterium *Citrobacter rodentium* (49). In addition, in our study, we found a potential correlation between the fungal taxon *Dioszegia* and the phenotype “anaerobic bacteria” across diets. *Dioszegia* has been proposed to be a “hub” fungal taxon; that is, it influences total host microbiome structure, and likely function, via its numerous interactions with other microbes that affect their colonization dynamics (e.g., by modulation of growth and/or diversity) (50). Altogether, these collective results highlight the potential importance of interkingdom interactions within the intestine for their role in human metabolic health.

The findings presented here provide a broader understanding of changes in the gut microbiome in association with high-fat diet and obesity. The role of microbes other than bacteria and of interkingdom interactions in the development of human health and disease should be a priority for future studies as the information gained could allow the discovery of novel targets for treatment and prevention of obesity.

## MATERIALS AND METHODS

### Animals.

All animal experiments were approved by the University of Minnesota Institutional Animal Care and Use Committee (IACUC), and all animals used in this study were treated and all animal experiments were performed in accordance with the University of Minnesota IACUC protocol 1605A33738. Male C57BL/6 mice were purchased from The Jackson Laboratory, housed according to treatment group, and cared for by Research Animal Resources at the University of Minnesota, which is accredited by the American Association for Accreditation of Laboratory Animal Care. Mice were fed either standard chow with 18% calories from fat and undetectable cholesterol (catalog no. 2018; Harlan Teklad, Madison, WI) (*n* = 9) or a high-fat diet with 60% calories from fat and 345 mg cholesterol/kg of body weight (catalog no. F3282; Bio-Serv, West Chester, PA) (*n* = 9) beginning at 8 weeks of age. Animals were euthanized at ~24 weeks of age by carbon dioxide inhalation.

### Metabolic phenotyping. (i) GTT and ITT.

The glucose tolerance test (GTT) and insulin tolerance test (ITT) were carried out in mice during week 16 of life, after fasting the mice overnight. Mice were intraperitoneally injected with 2 g/kg d-glucose for GTT or 0.75 U/kg insulin for ITT. Blood glucose levels were measured with a standard glucometer before and after injection at 30, 60, 90, and 120 min.

### (ii) Oxygen consumption.

Indirect calorimetry of mice was performed on mice of ~16 weeks of age (*n* = 6 mice/group) to measure oxygen consumption (vO_2_). Before measurement, mice were trained to habituate to metabolic cages for 3 days and maintained in individual chambers with free access to food and water in the animal facility with 12-h light/dark cycles. The vO_2_ measurement was recorded every 4 min for 2 days and normalized to body weight using Oxymax software (Columbus Instrument, Columbus, OH).

### (iii) Metabolic biomarker assays.

Blood samples were collected from animals at the time of sacrifice (~24 weeks of age), and metabolic biomarker levels were measured using commercially available enzyme-linked immunosorbent assay (ELISA) kits for plasma insulin and adiponectin (EMD Millipore, Billerica, MA) and a fluorimetric assay for total serum cholesterol (Cayman Chemical, Ann Arbor, MI). The L-type TG M kit and NEFA-HR(2) kit (Wako Diagnostics, Richmond, VA), which utilize an enzymatic colorimetric method, were used to measure serum triglycerides and free fatty acids, respectively.

### Fungal ITS2 amplicon generation and sequencing.

DNA was extracted from fecal pellets obtained from mice during week 16 of life and from three separate samples of both the high-fat diet and the standard mouse chow using the PowerSoil DNA isolation kit (Mo Bio Laboratories, Inc., Carlsbad, CA) according to the manufacturer’s protocol for difficult DNA extractions: samples were incubated at 75°C for 5 min after suspension in solution C1, and isolated DNA was suspended in 100 µl of water and stored at −20°C until use. Amplicons targeting the fungal internal transcribed spacer region 2 (ITS2) were generated in triplicate for all samples according to protocols previously described (15) with the following modifications. ITS2-specific oligonucleotide primers (15) were modified to include a barcode of 6 bp for both the forward and the reverse primers; this dual barcoding improves the amount of multiplexing that can be performed on a single sequencing run. In addition, degenerate base pairs were included on the 5′ end of each primer; this is thought to improve the quality of sequencing on MiSeq instruments due to increased length diversity and reduction in base pair homogeneity during photo acquisition during sequencing (University of Minnesota Genomics Center, personal communication). Once generated, amplicons were cleaned using the QIAquick PCR purification kit (Qiagen, Germantown, MD) as previously described (15) and eluted into water. Cleaned amplicons were quantified using a Qubit 2.0 fluorometer (Invitrogen, Eugene, OR) using the Qubit dsDNA HS assay kit (Invitrogen). Equal amounts of DNA from each sample were pooled in water and submitted to the University of Minnesota Genomics Center for DNA library preparation using the TruSeq Nano kit and sequencing using the Illumina MiSeq platform.

### Bacterial 16S rRNA gene amplification and sequencing.

Fecal pellets were collected from mice during week 16 of life and submitted to the University of Minnesota Genomics Center for DNA extraction, generation of 16S amplicons, and sequencing. Briefly, DNA was extracted using the PowerSoil DNA isolation kit, followed by amplification of the V6 region of the 16S rRNA gene using standard methods developed by the Earth Microbiome Project (51). DNA libraries were generated from the resulting amplicons using the Illumina TruSeq Nano kit (Illumina, San Diego, CA), and amplicons were then sequenced by the Illumina MiSeq platform using the 2- by 300-bp paired-end V3 kit (Illumina).

### Microbial community analysis. (i) Fungi.

Fungal sequences were paired, filtered for quality, and aligned using an experimental new aligner, BURST (52), which was developed to perform optimal exhaustive alignment and interpolate the taxonomy annotations for all reference database matches for each query read. For instance, if a given query read matched with a similar alignment score to 5 references, all of which have the same genus name but conflicting species names, BURST would annotate that read at the genus level without overconfidently committing to a single species for which there was conflicting sequence evidence. To circumvent issues related to the uncurated nature of the UNITE database (53, 54), ITS2 sequences were aligned to the RefSeq v81 Targeted Locus Project ITS region database (53), which contains a highly curated set of approximately 5,500 full-length fungal ITS markers that have been sequenced and characterized using a consistent protocol. Replicate samples were pooled bioinformatically prior to analyses to provide a more robust representation of taxa within the samples (15). There was a mean of 19,470 fungal sequences per sample, with a range of 2,435 to 71,050 reads per sample. The taxonomy table was rarefied to 2,435 sequences to perform diversity analyses. Alpha-diversity analyses were performed using Shannon, Chao1, and unique observed species indices. Beta-diversity analyses were performed using Bray-Curtis distance metrics, as implemented in Quantitative Insights Into Microbial Ecology 1.8.0 (QIIME [51]).

### (ii) Bacteria.

The sequence data were processed in QIIME. Data were quality filtered and demultiplexed using default parameters. Sequences were assigned to 97% identity operational taxonomic units (OTUs) by comparison to the Greengenes reference database 13_8 (55) using UCLUST (56). The OTU table was then rarefied to 90,851 sequences to perform diversity analyses. Alpha-diversity analyses were performed using Shannon and phylogenetic diversity indices. Beta-diversity analyses were performed using unweighted and weighted UniFrac (57) distance metrics, as implemented in QIIME. The relative abundances of 558 level 3 KEGG modules were predicted from the OTU table using BugBase (28).

### (iii) Statistical analyses.

Principal-component analysis (PCoA), permutational analysis of variance (PERMANOVA) tests, and Procrustes analysis were performed in QIIME. Comparison of taxonomic count data and KEGG modules between fecal samples collected from mice on a high-fat diet and those collected from mice on a normal diet was performed using the Mann-Whitney U test with false discovery rate (FDR) correction for multiple testing. Box plots, bee swarm plots, heat maps, randomForest analyses, and two-dimensional PCoA plots were generated using R (58). We also performed Spearman correlations between relative abundances of fungal and bacterial profiles and between relative abundances of fungal taxa and bacterial KEGG modules and developed a permutation-based test of the Spearman correlation test statistic with 1,000 permutations to test for conserved correlations between diets and BugBase phenotypes using R. We plotted a network of fungal and bacterial pairs that significantly correlated with an FDR-corrected *P* value of <0.05 with Cytoscape (59).

### qPCR.

Quantitative real-time PCR (qPCR) analysis was performed on a limited number of samples as an additional method to characterize fungal presence in the samples. All fecal DNA samples were subjected to qPCR (Roche LightCycler 480; Roche, Indianapolis, IN) with universal fungal primers. In addition, a subset of five fecal samples as well as chow samples was subjected to qPCR using fungal species-specific primers. qPCR protocols were performed as previously described by our research group (15) and included negative (no-template) controls to study the potential for contamination of samples with exogenous fungal DNA.

### Availability of data.

Sequencing results are available in the Sequence Read Archive (SRA) database at NCBI under BioProject ID PRJNA353013. R scripts are posted at http://galelab.umn.edu/.
