# Supplementary figures and images for: High-Fat Diet Changes Fungal Microbiomes and Interkingdom Relationships in the Murine Gut
Source: mSphere. 2017 Oct 11;2(5):e00351-17. doi: 10.1128/mSphere.00351-17 (PMC5636226; doi:10.1128/mSphere.00351-17)

PD whole tree: Diet

a

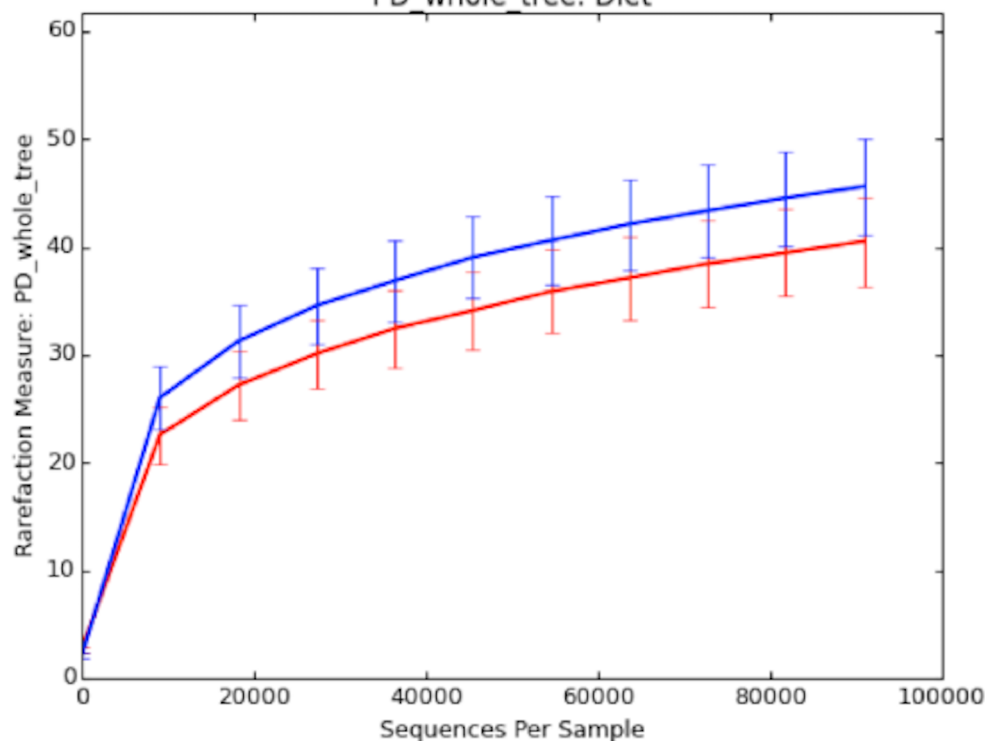

shannon: Diet

b

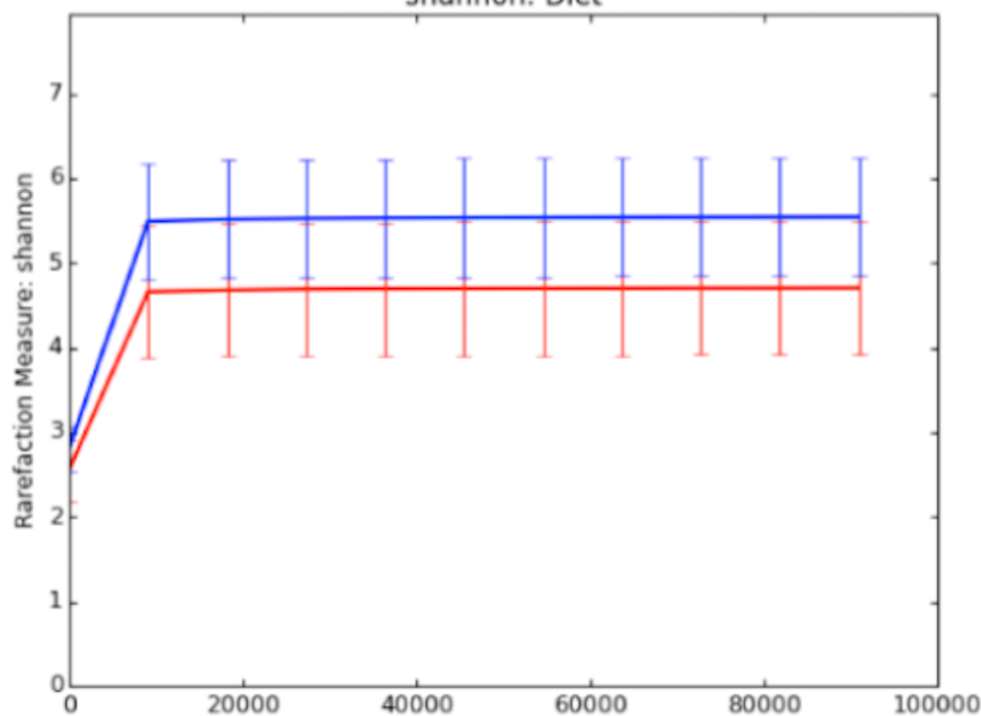

Supplement: FIG S1 [file sph005172381sf1.pdf]

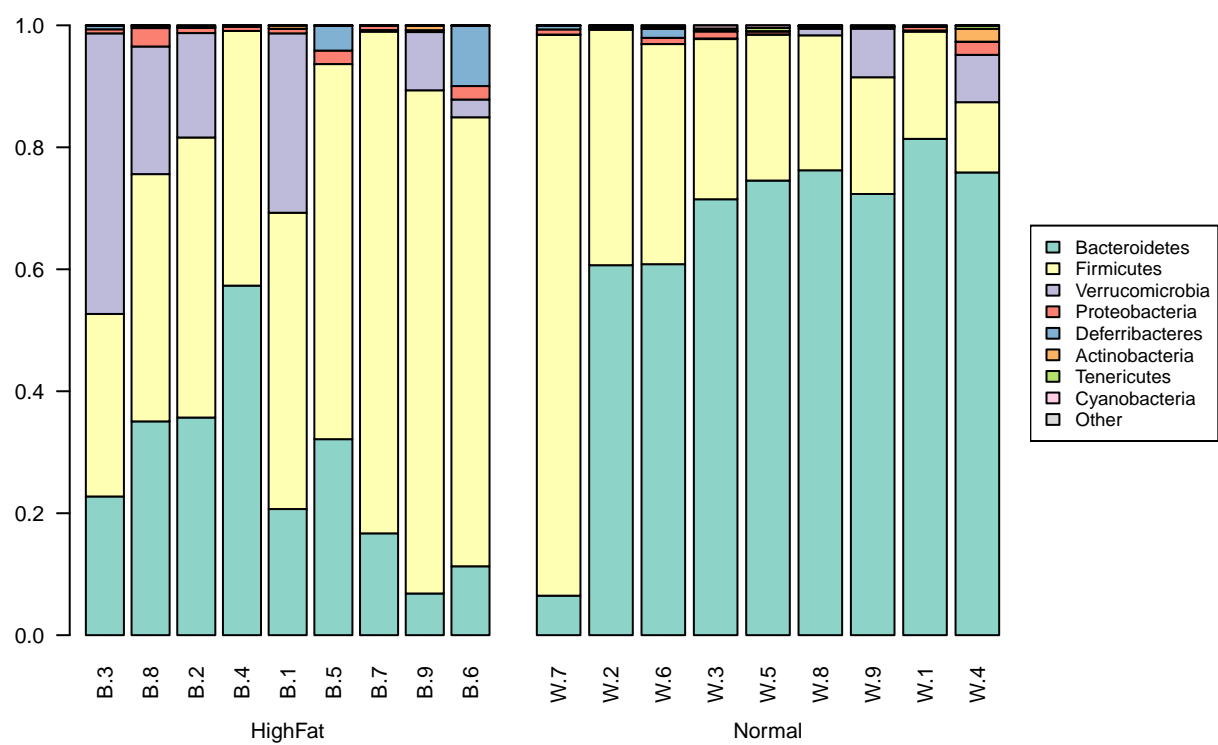

Supplement: FIG S3 [file sph005172381sf3.pdf]

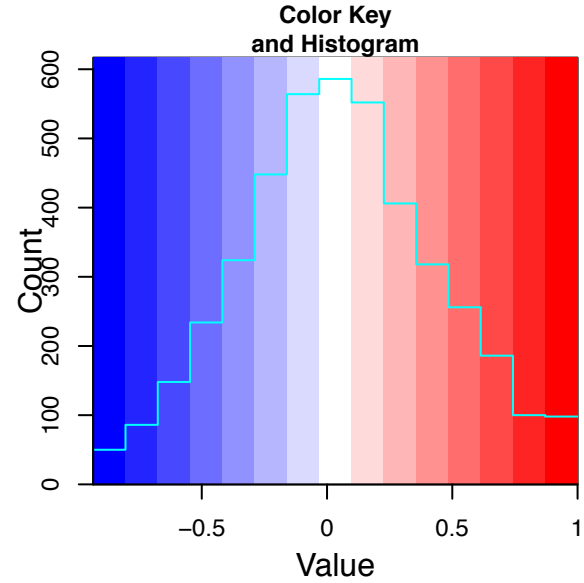

a

correlation 16S ND WT

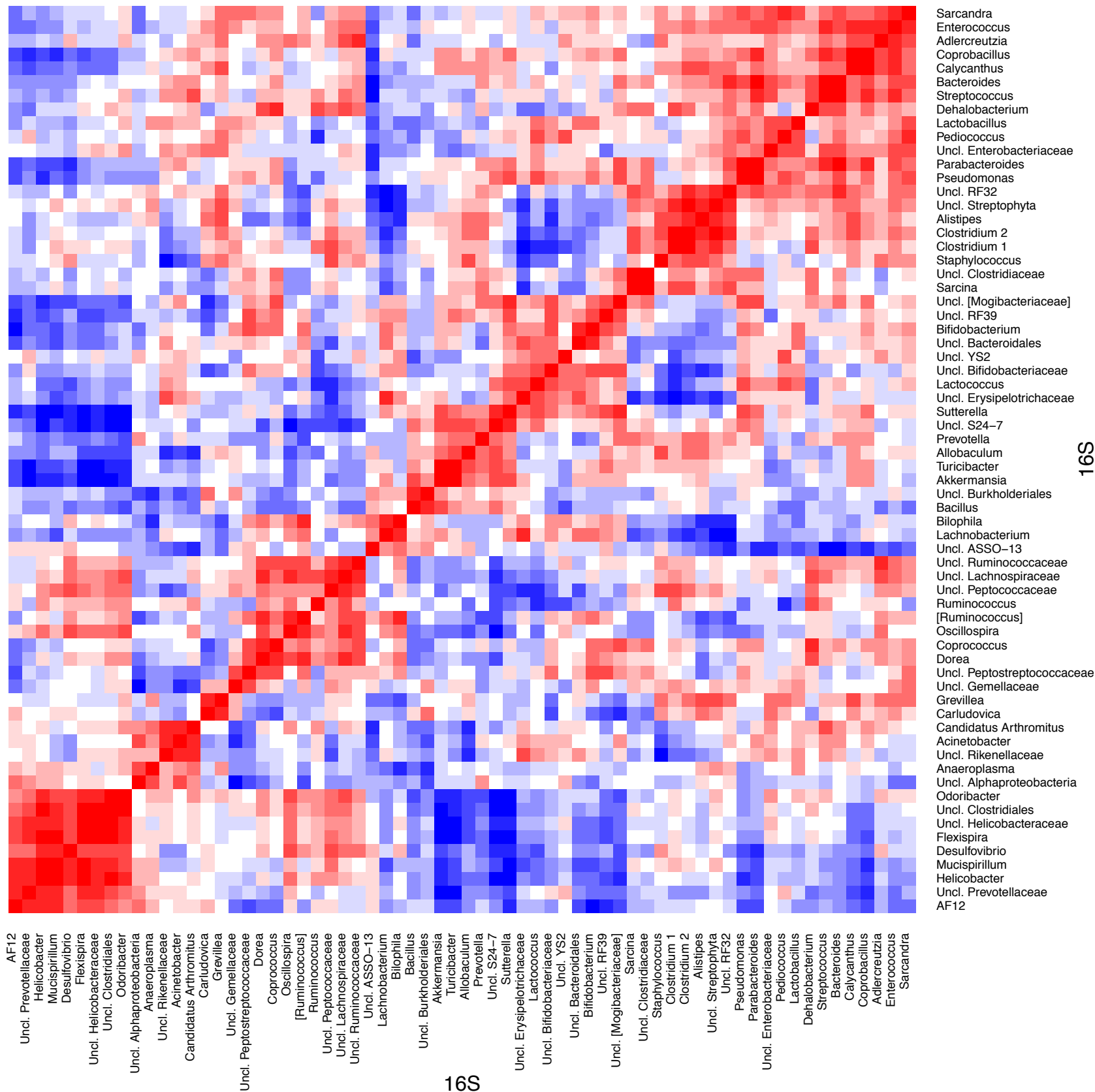

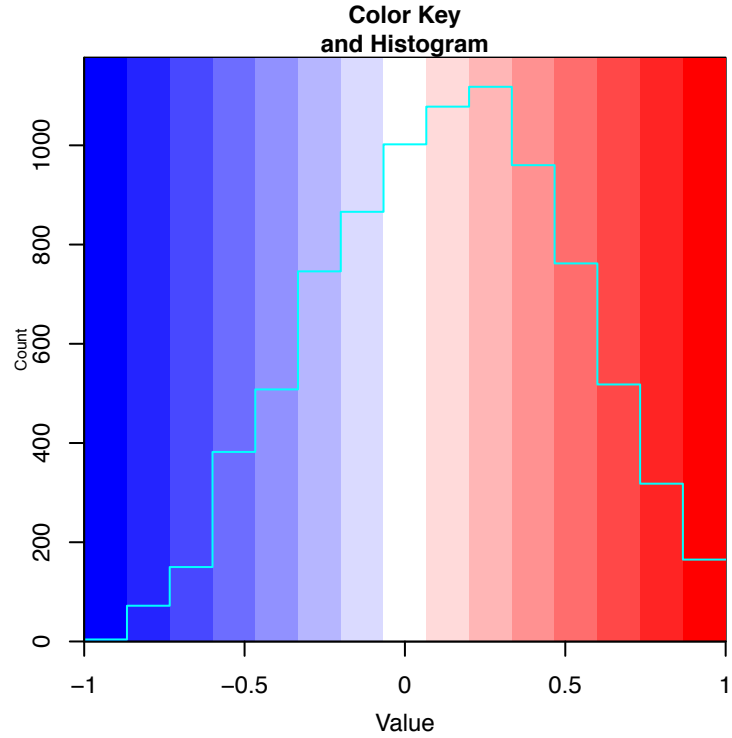

**b**

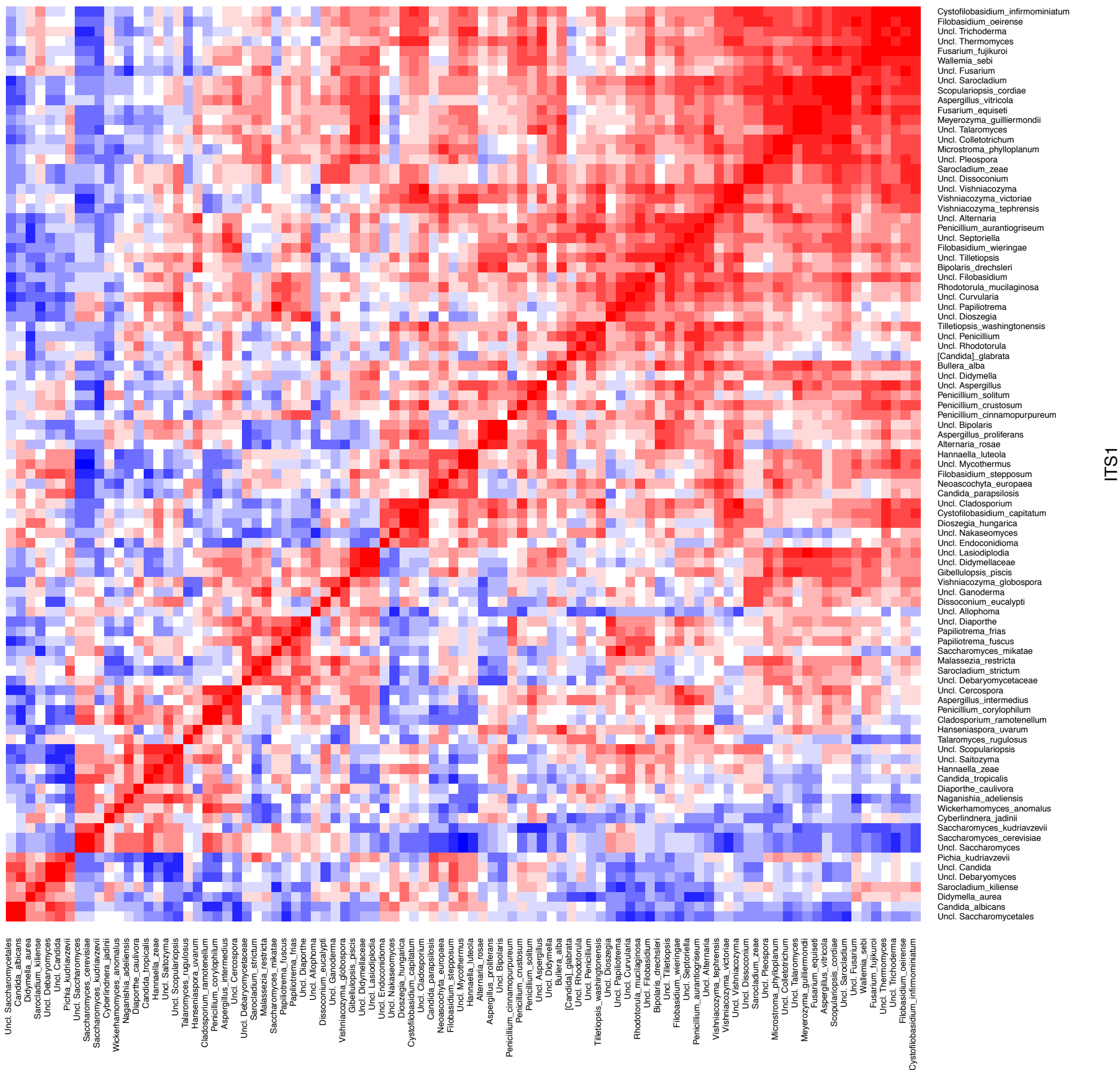

ITS1

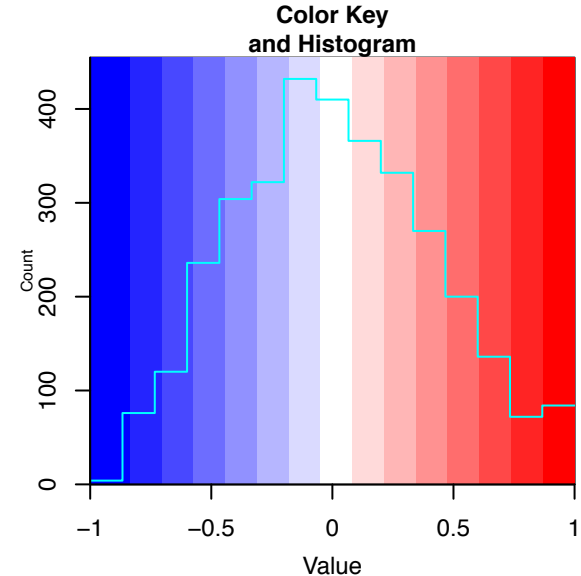

correlation 16S HF WT

C

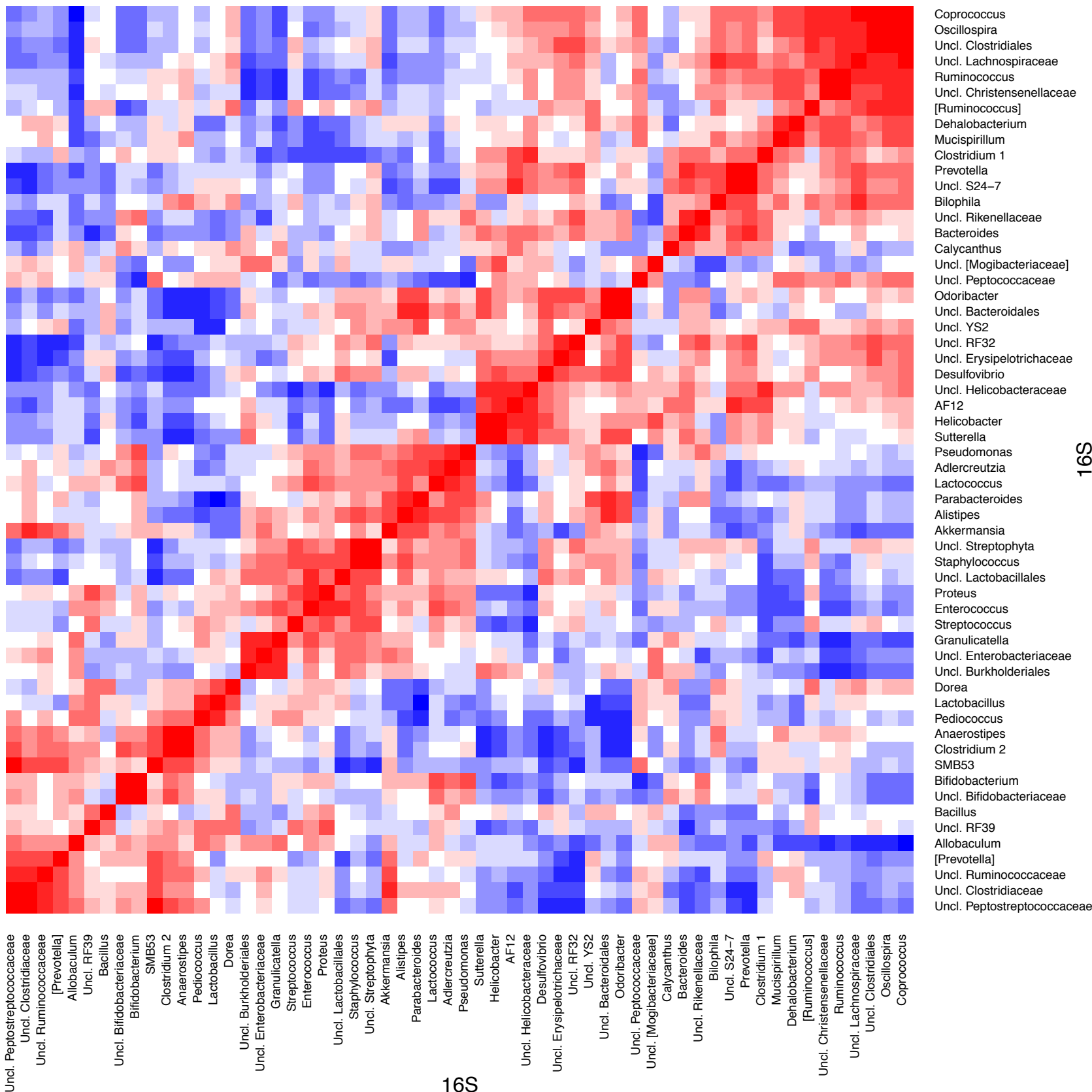

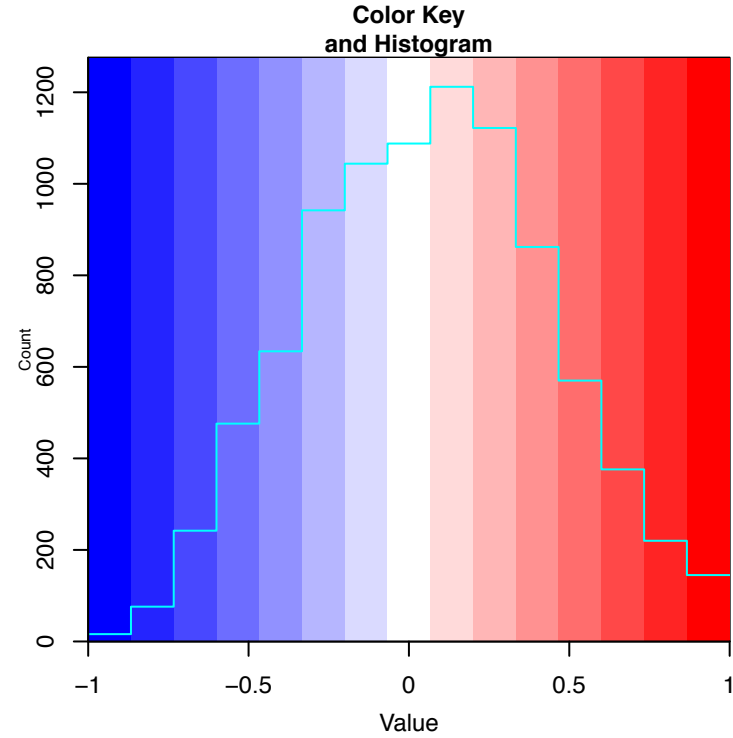

correlation ITS1 HF WT

d

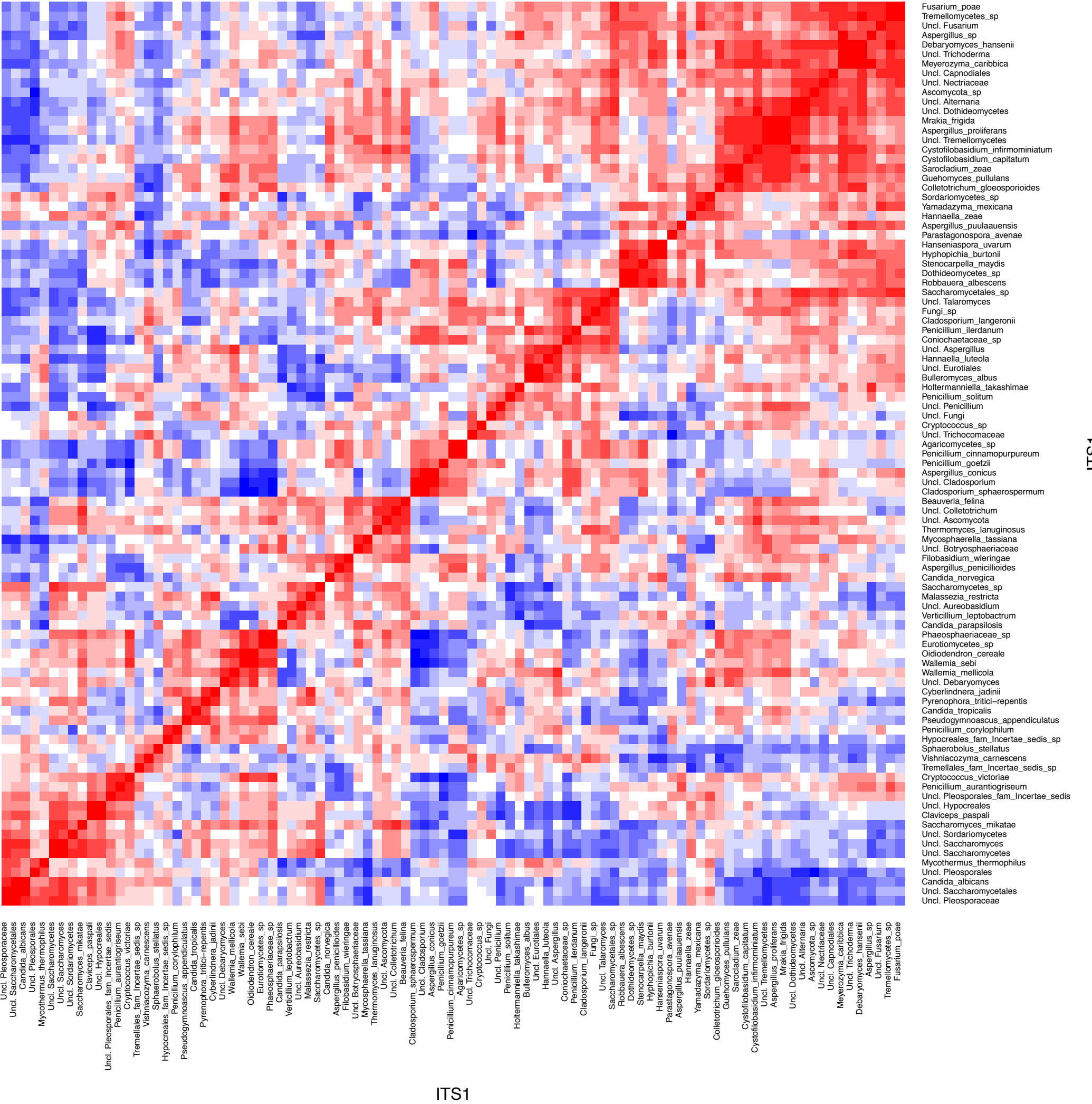

Supplement: FIG S6 [file sph005172381sf6.pdf]

C

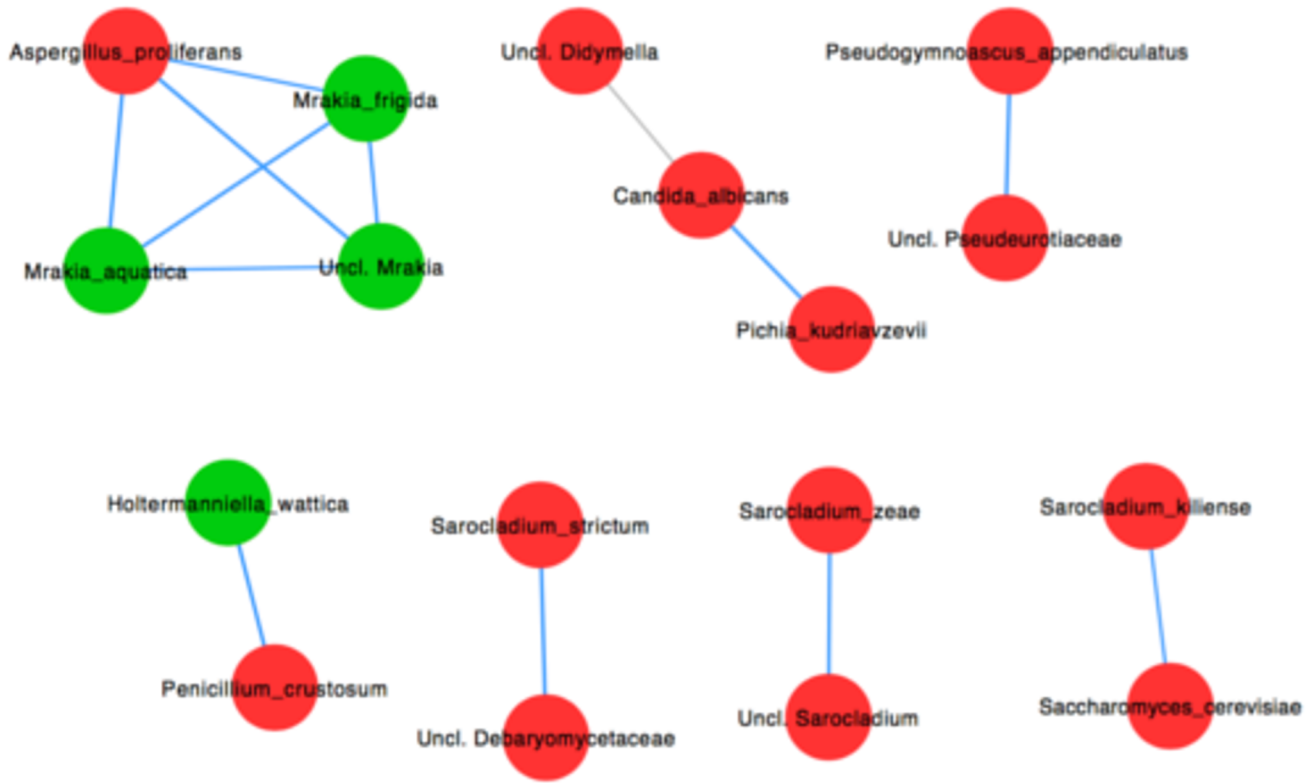

d

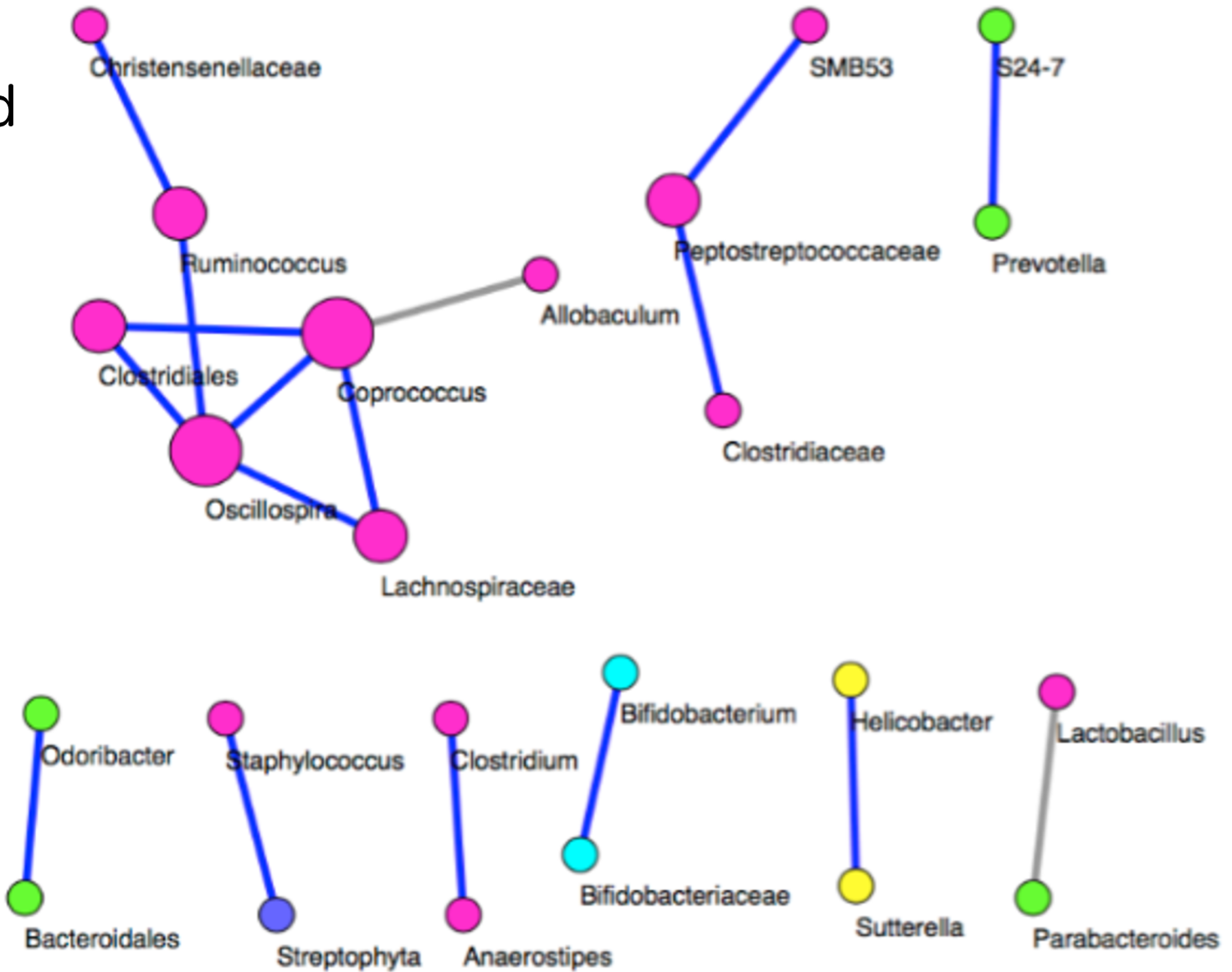

Supplement: FIG S7 [file sph005172381sf7.pdf]

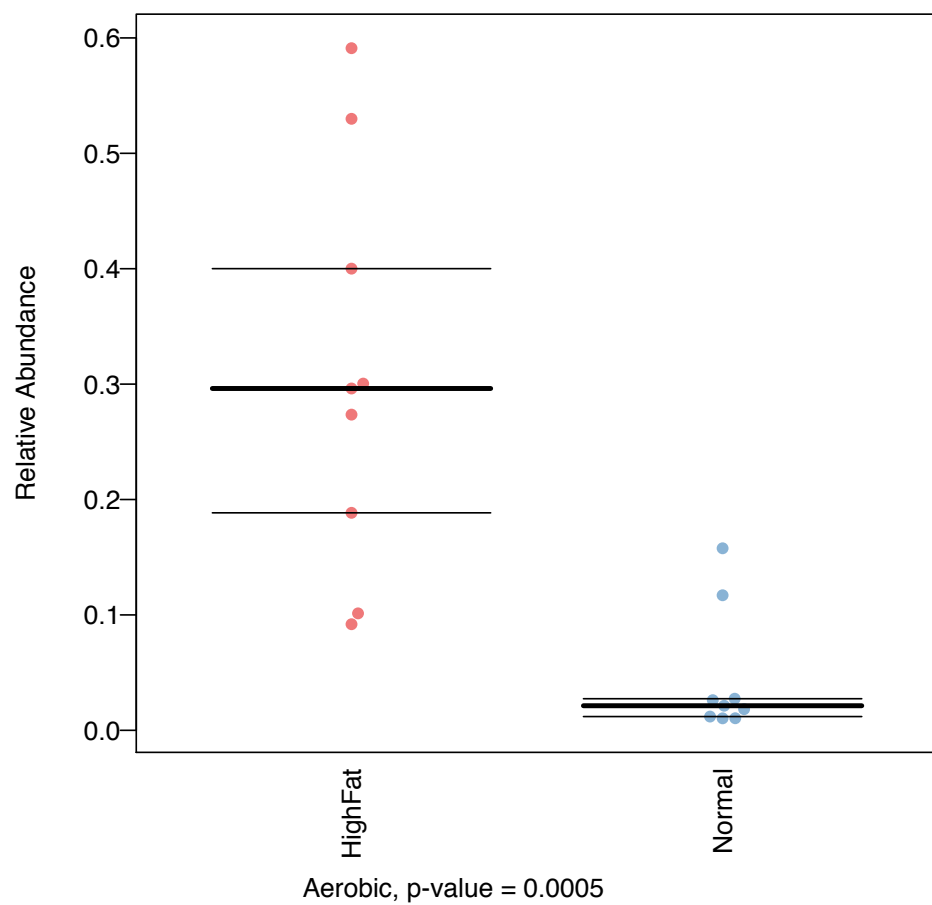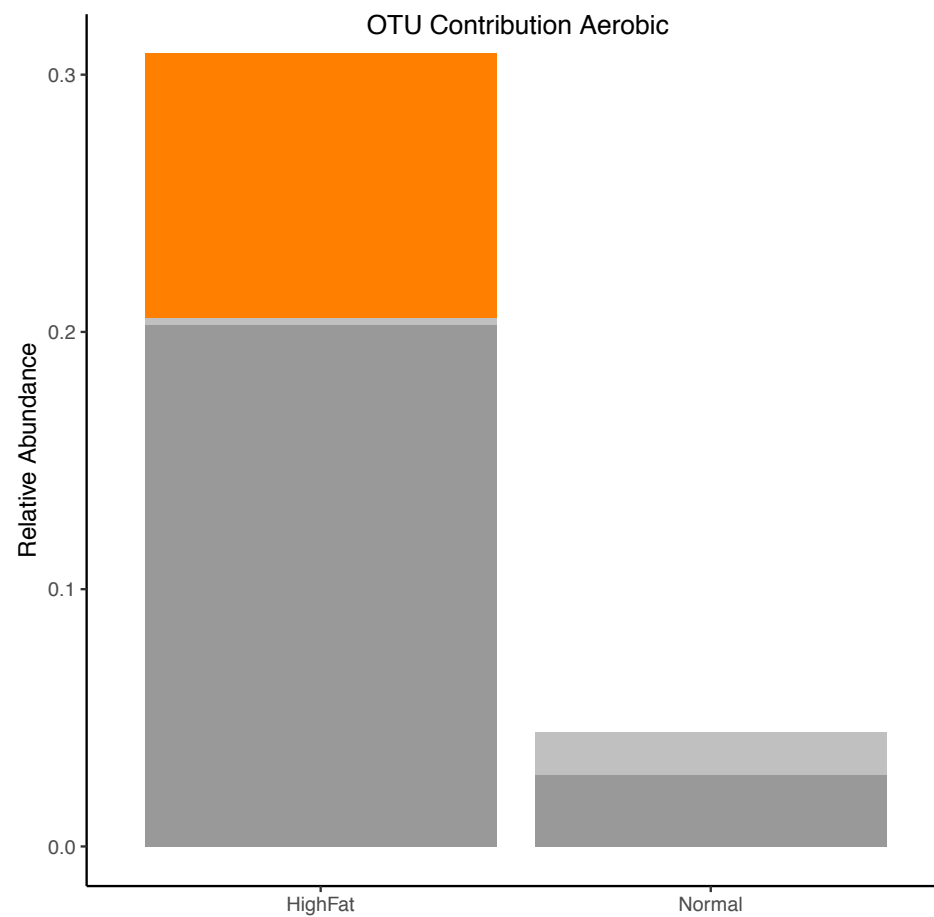

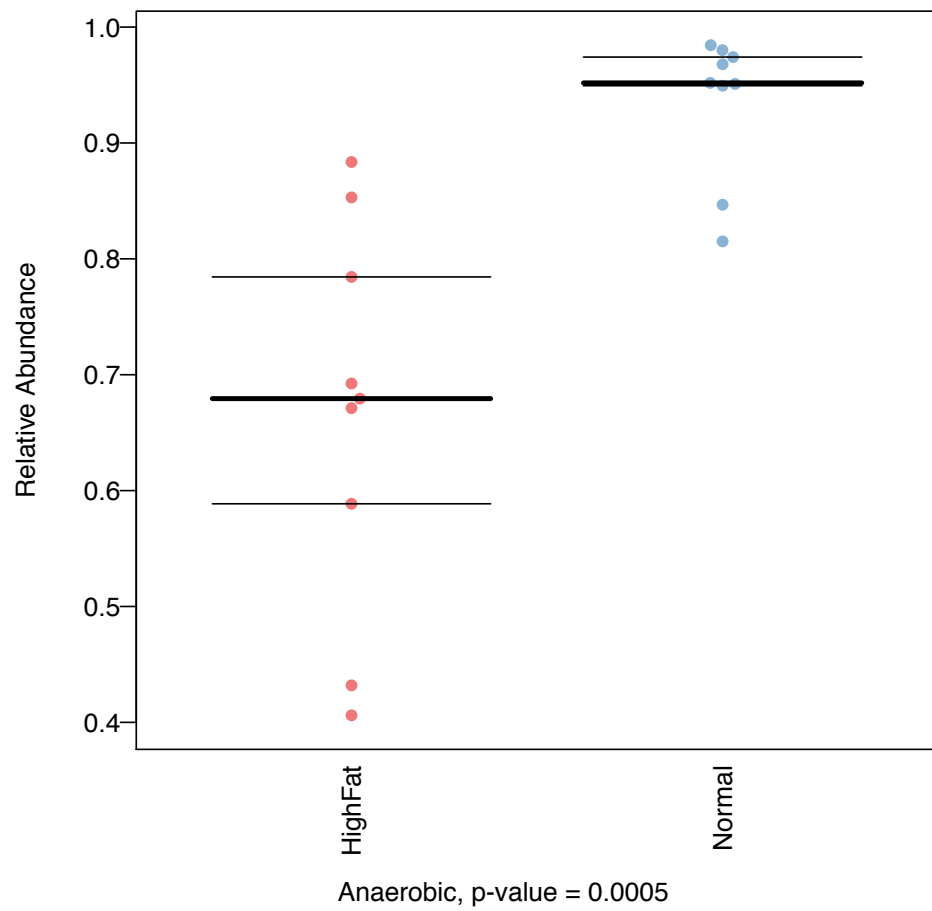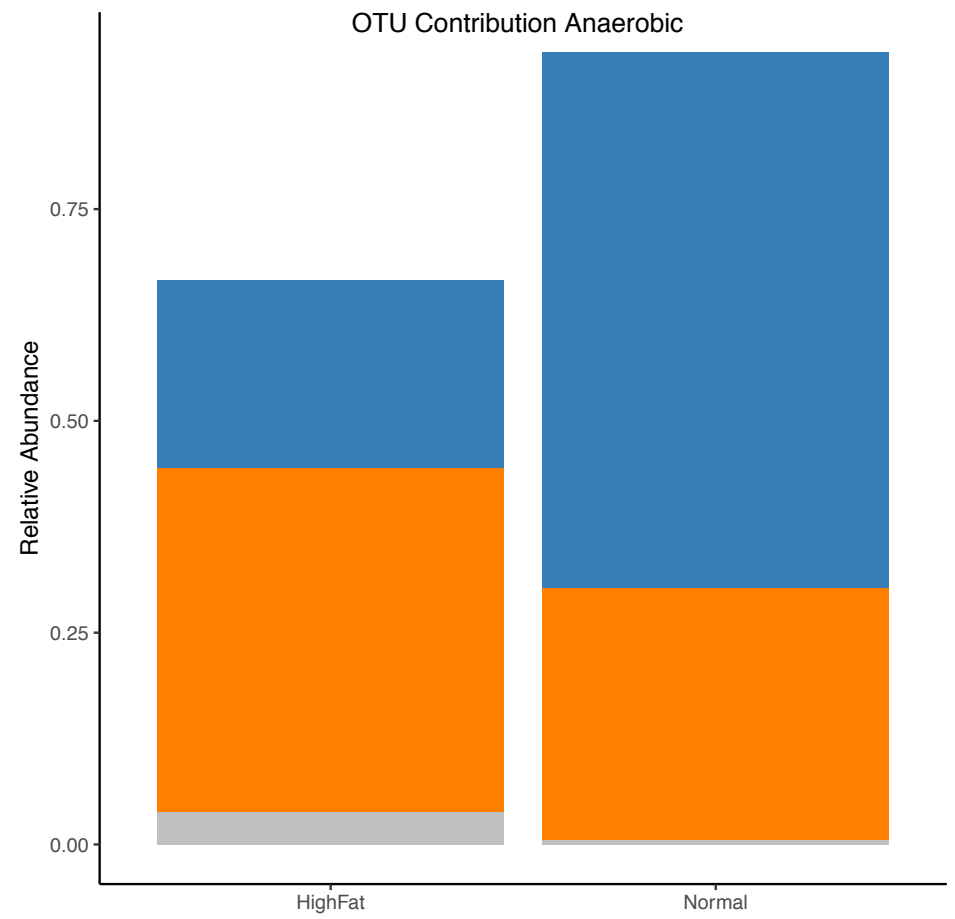

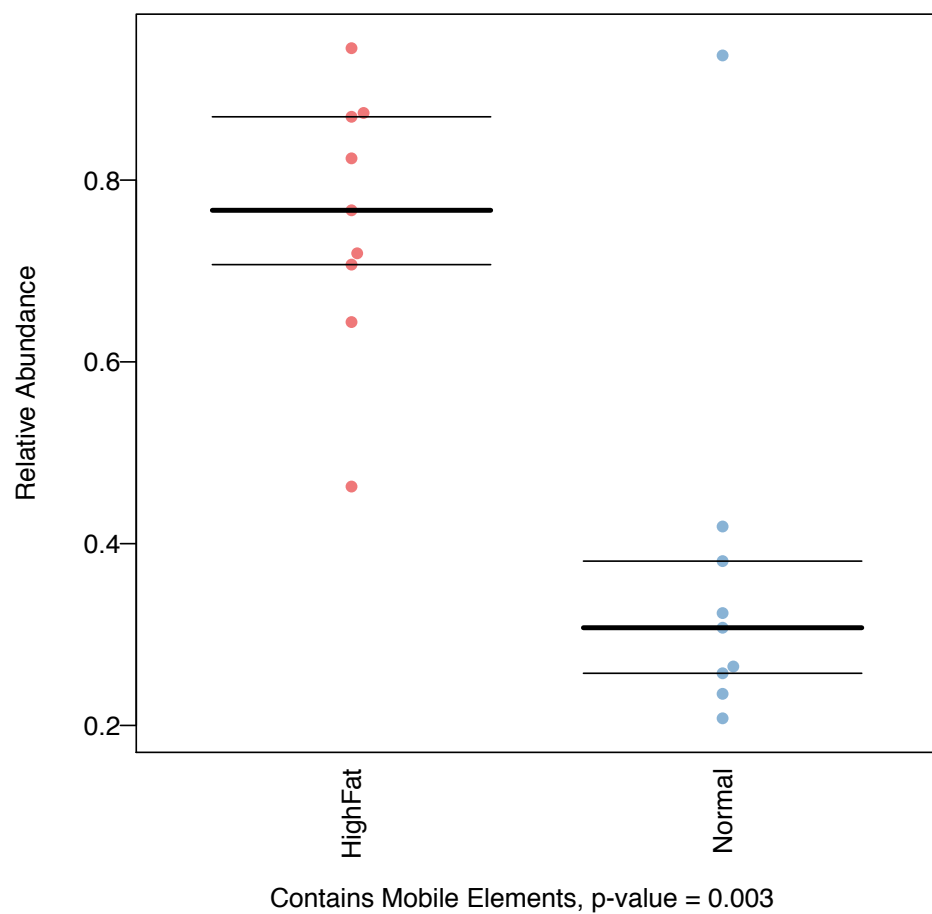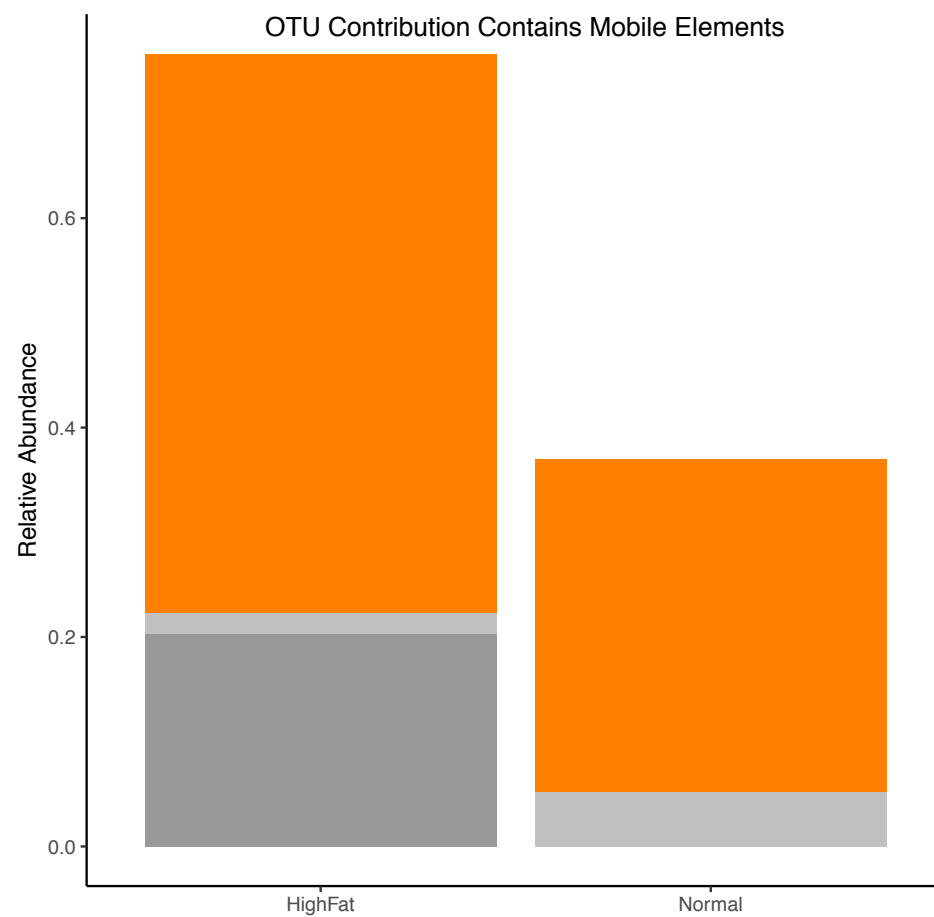

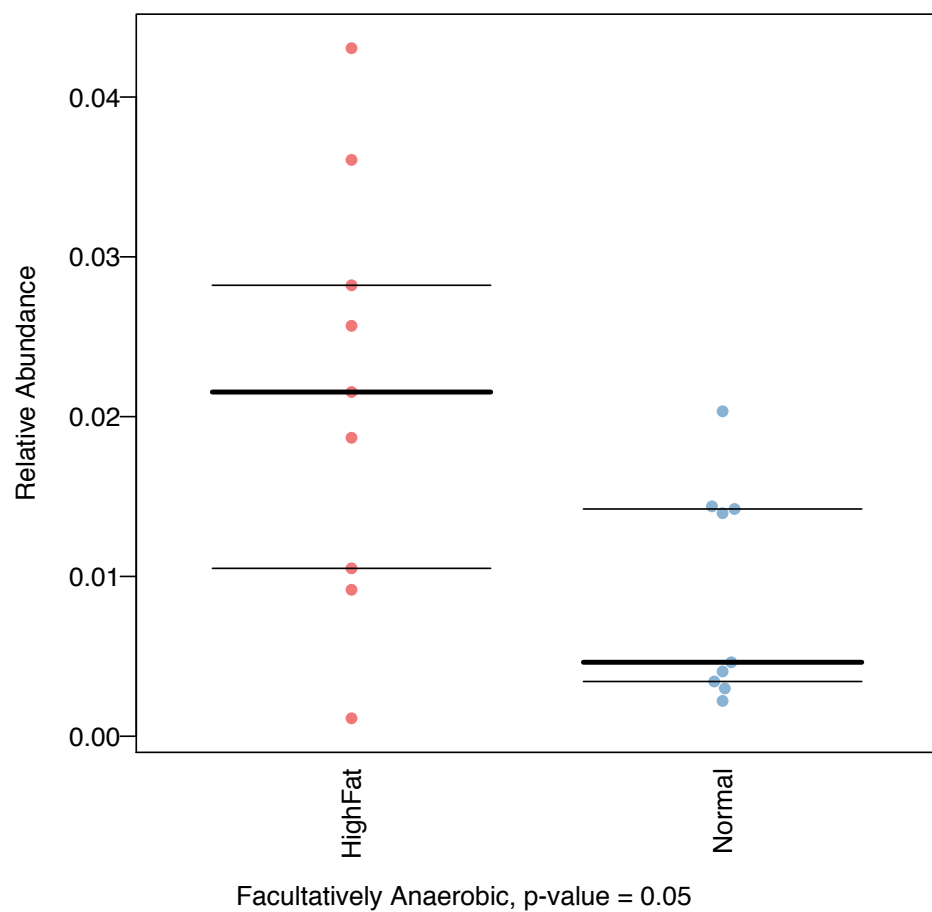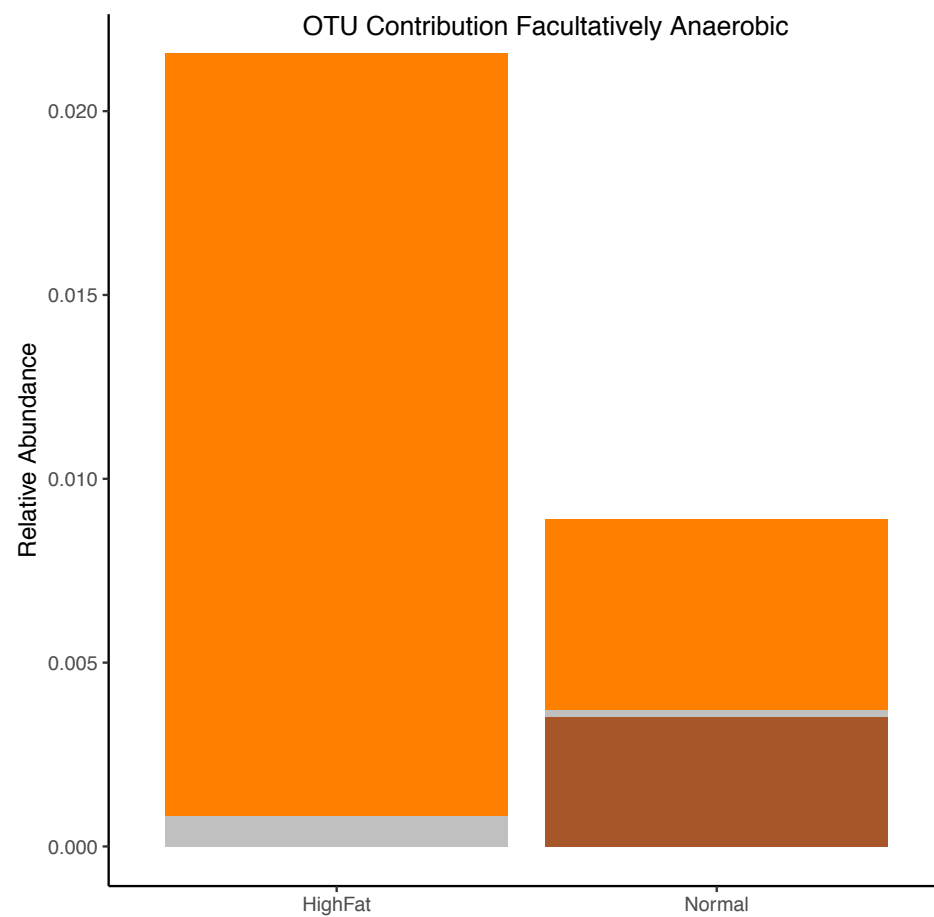

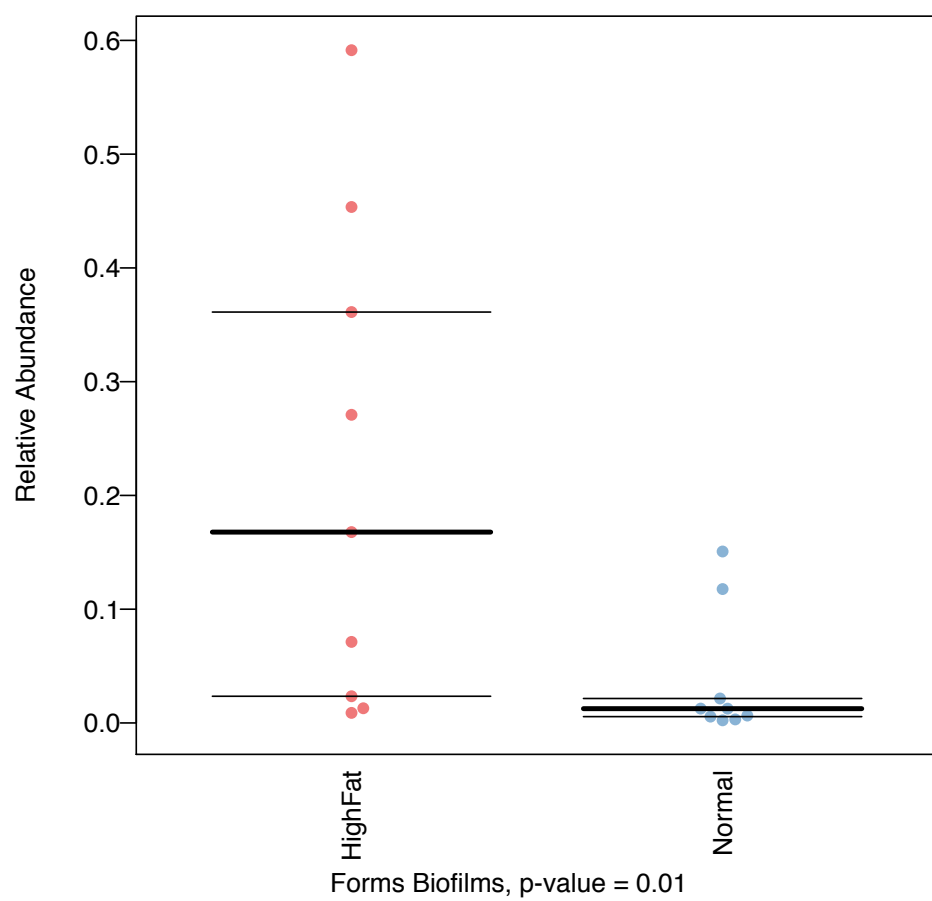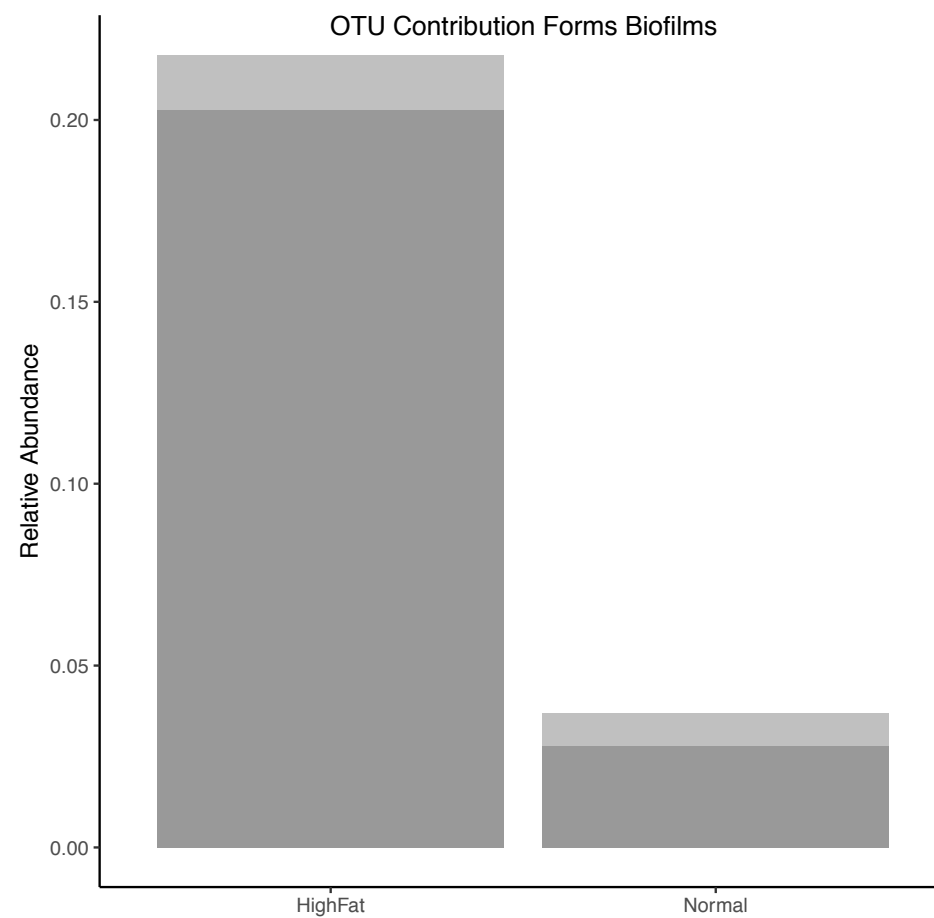

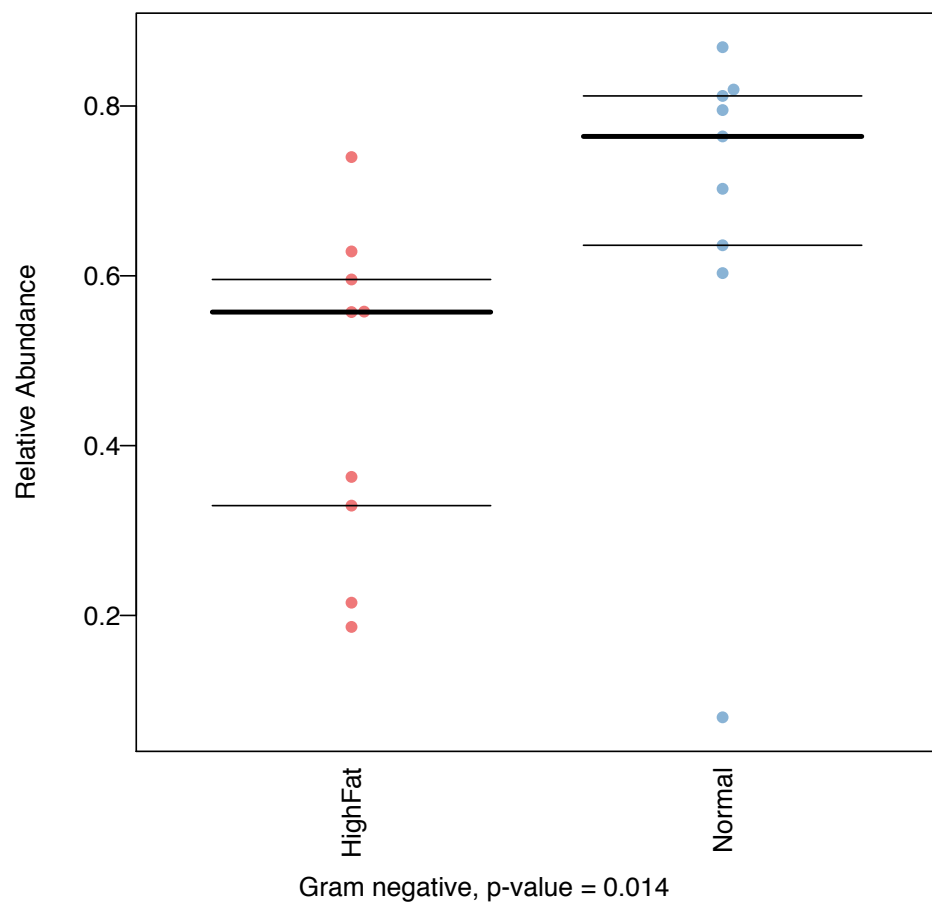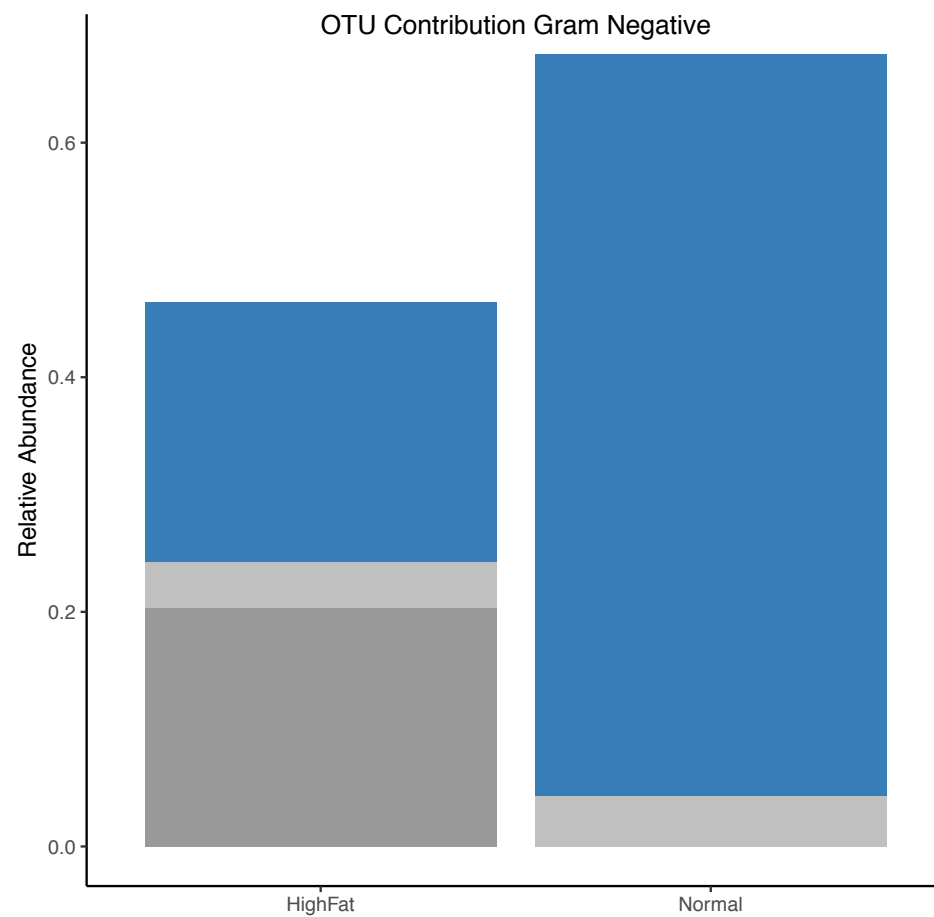

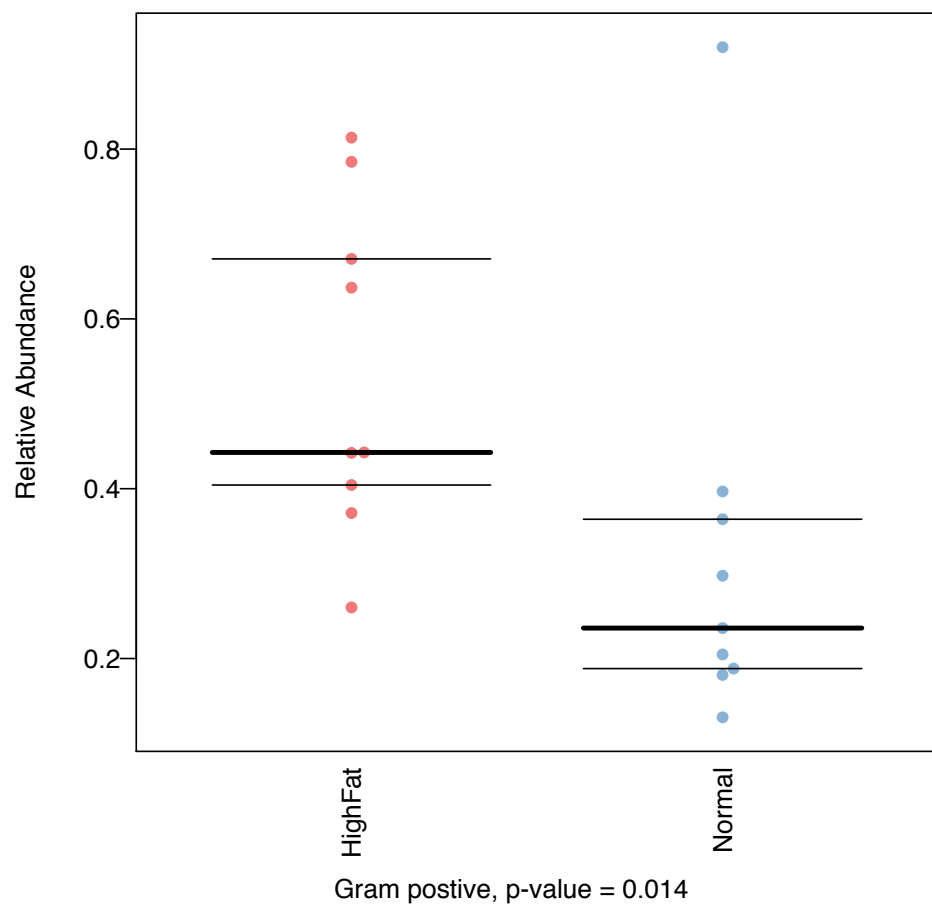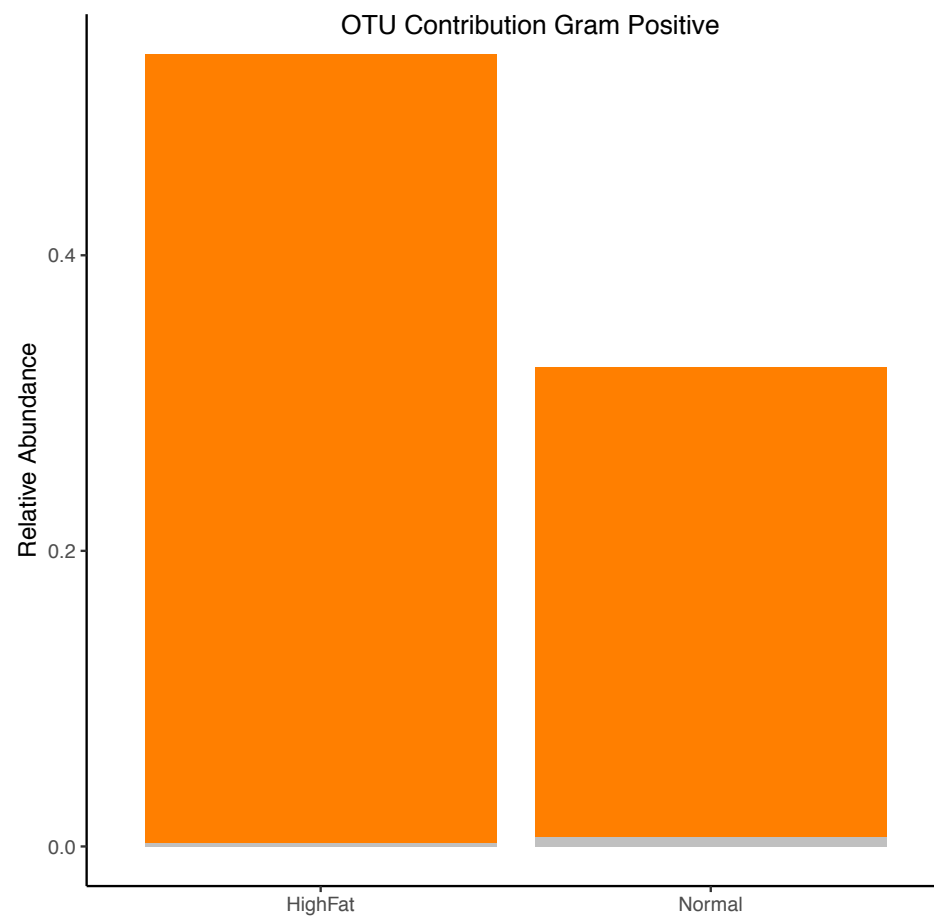

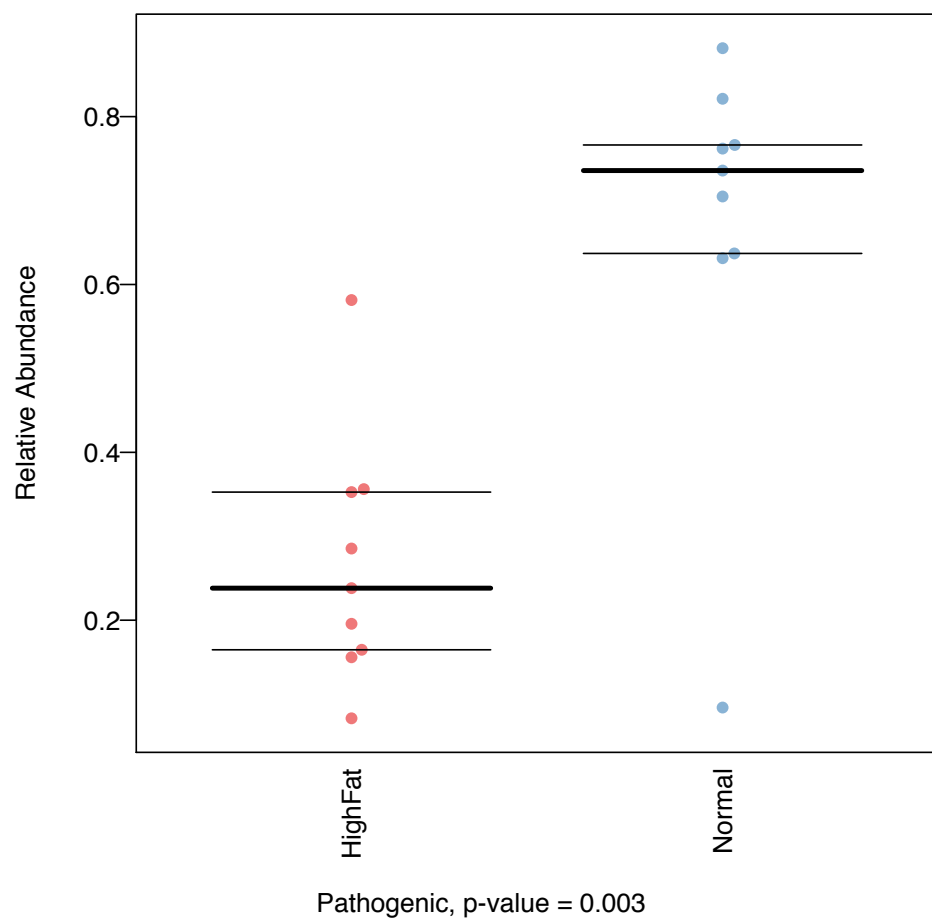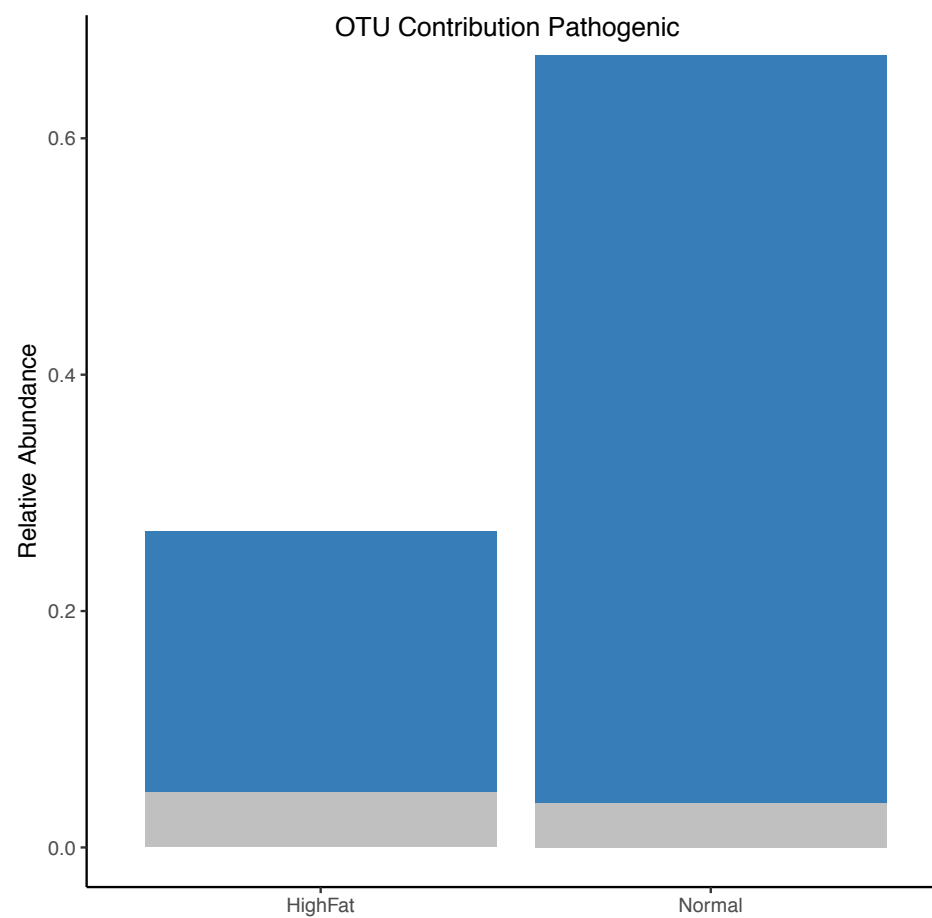

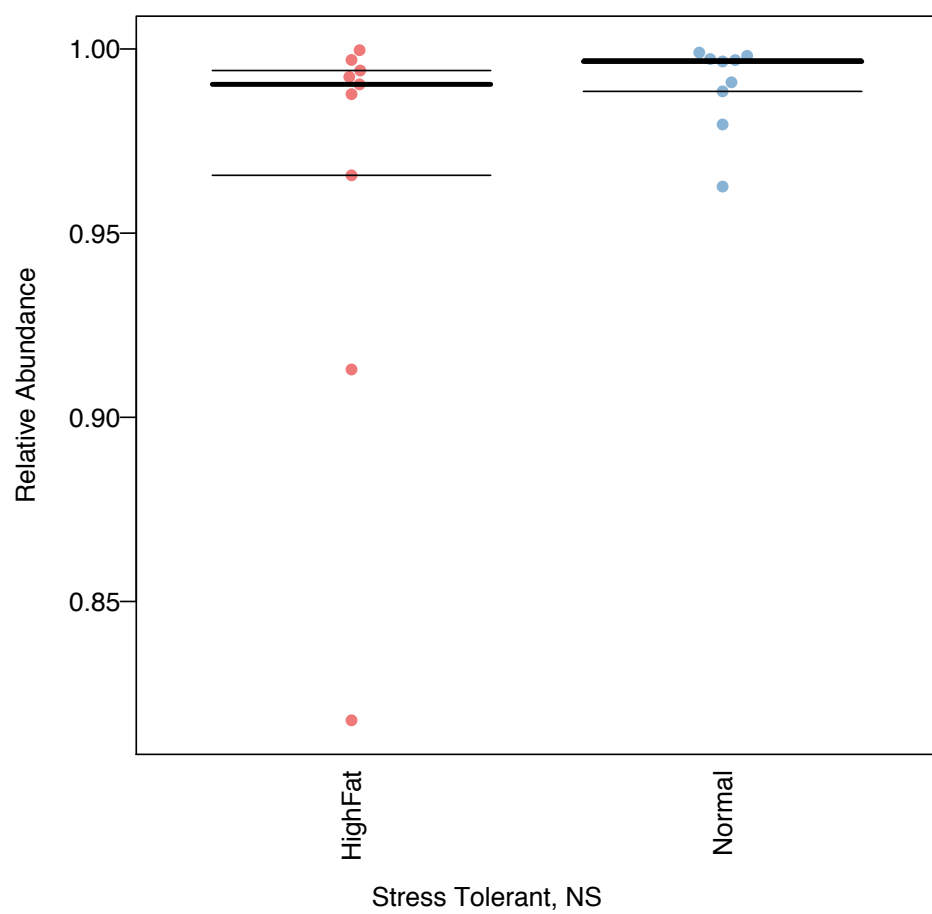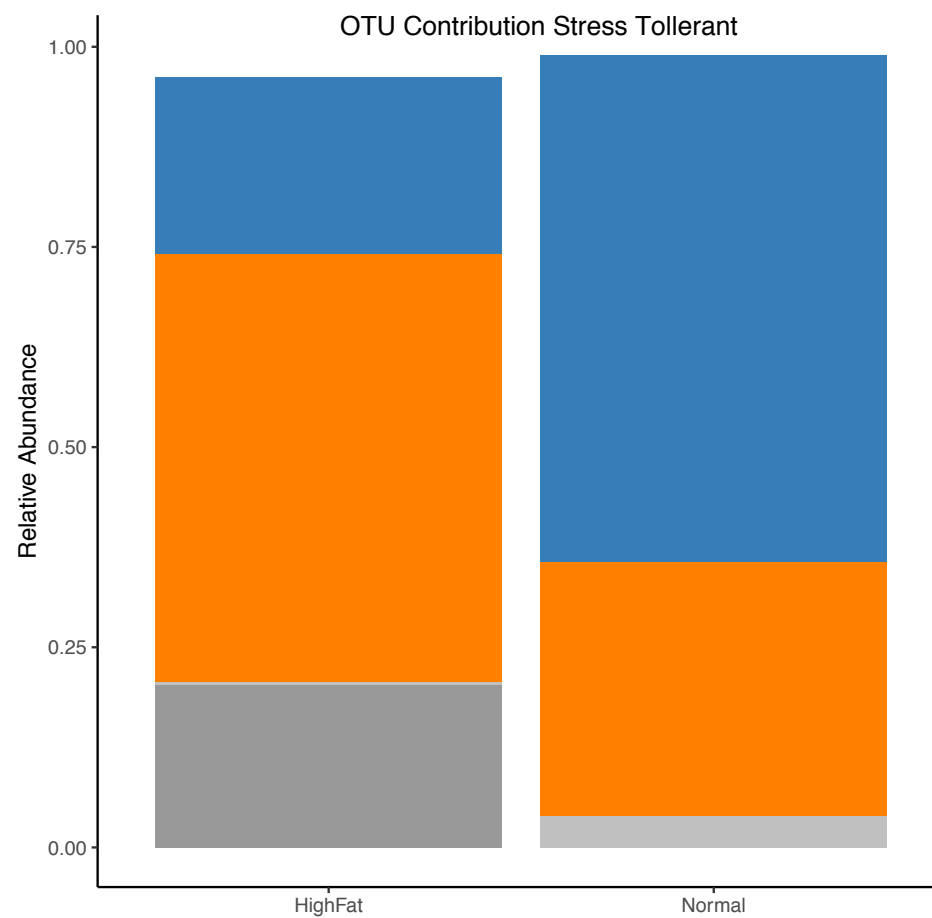

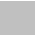 Other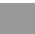 Verrucomicrobia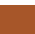 Tenericutes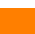 Firmicutes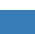 Bacteroidetes

Supplement: FIG S8 [file sph005172381sf8.pdf]

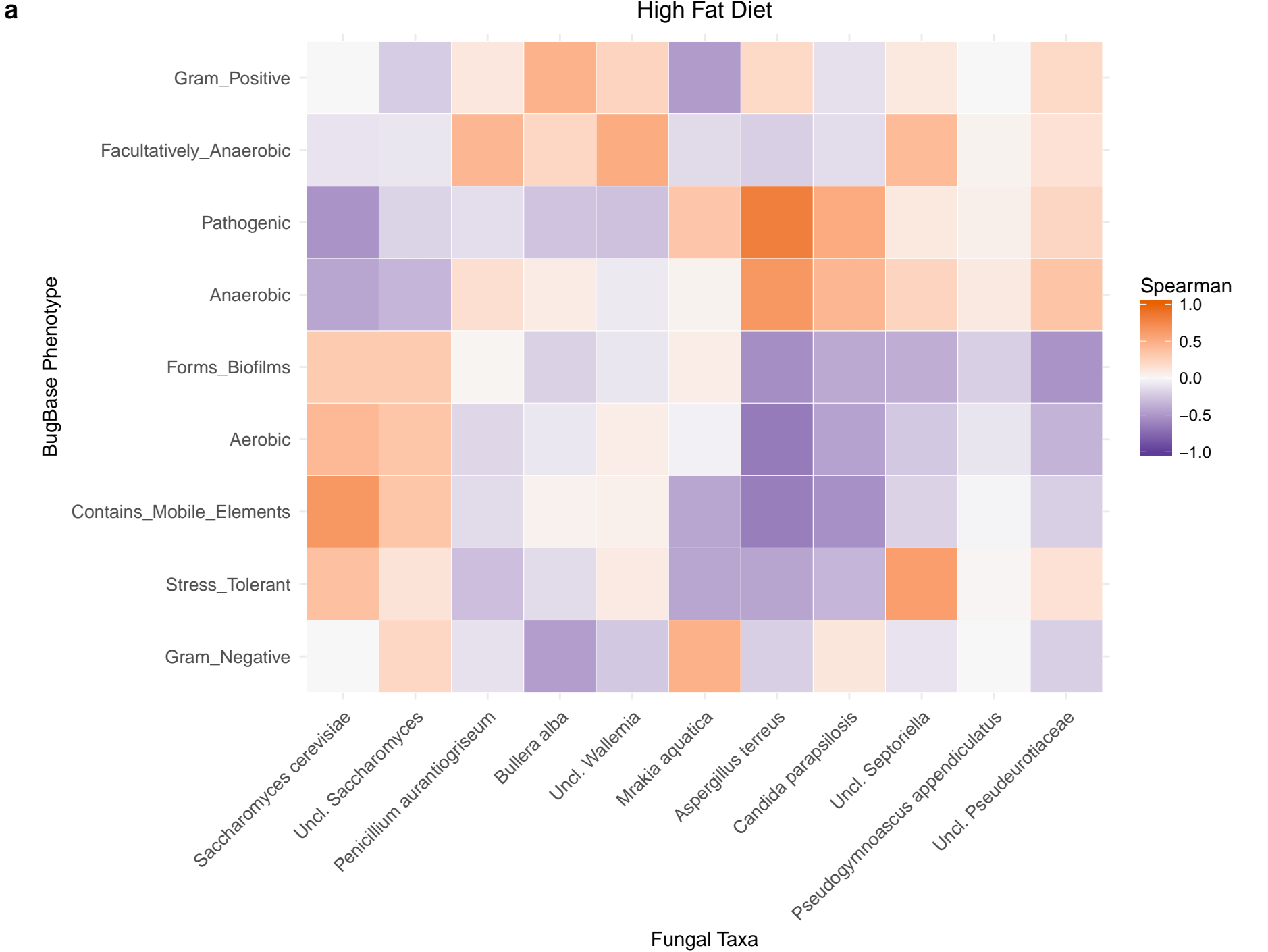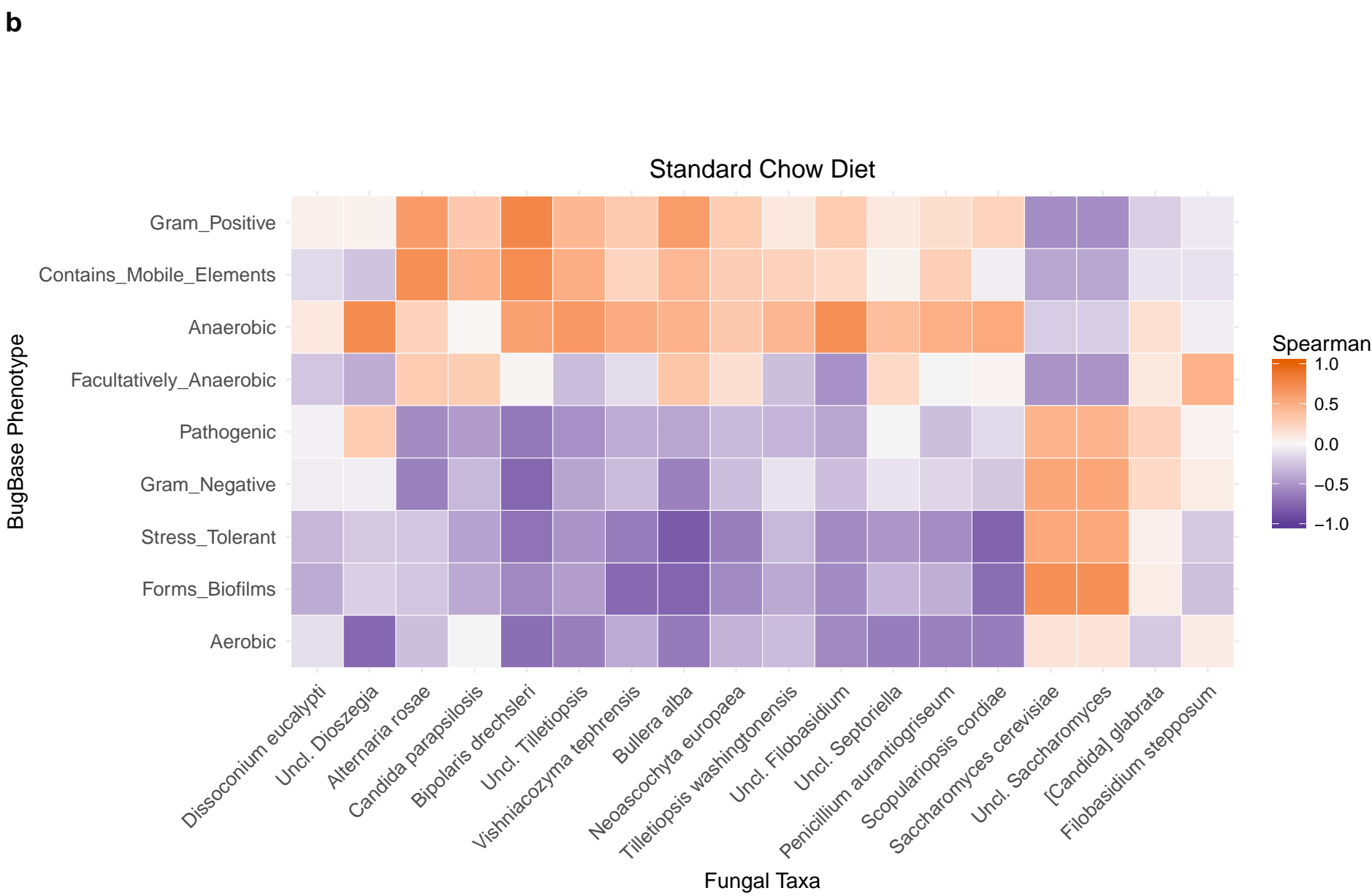

Supplement: FIG S9 [file sph005172381sf9.pdf]
