# Supplementary material for: High-Fat Diet Changes Fungal Microbiomes and Interkingdom Relationships in the Murine Gut
Source: mSphere. 2017 Oct 11;2(5):e00351-17. doi: 10.1128/mSphere.00351-17 (PMC5636226; doi:10.1128/mSphere.00351-17)
Supplement: FIG S2 [file sph005172381sf2.pdf]

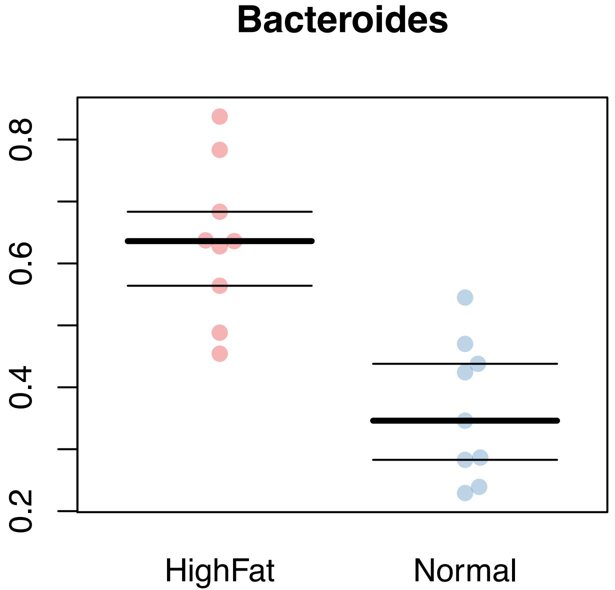
Bacteroides
0.2
0.4
0.6
0.8
HighFat
Normal

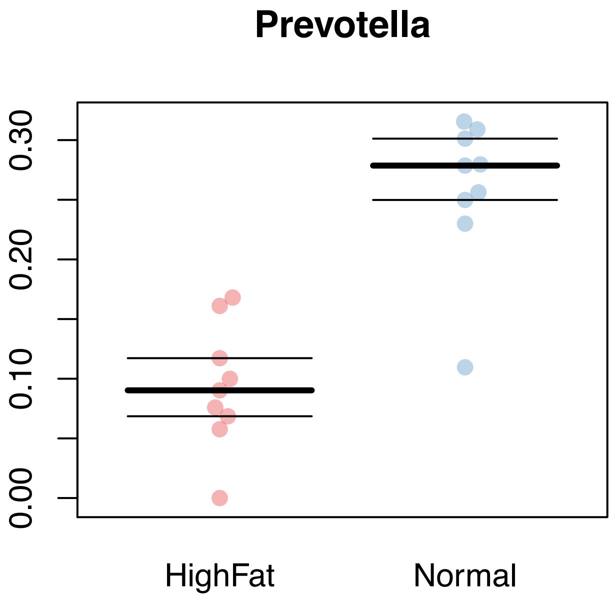
Prevotella
0.00
0.10
0.20
0.30
HighFat
Normal

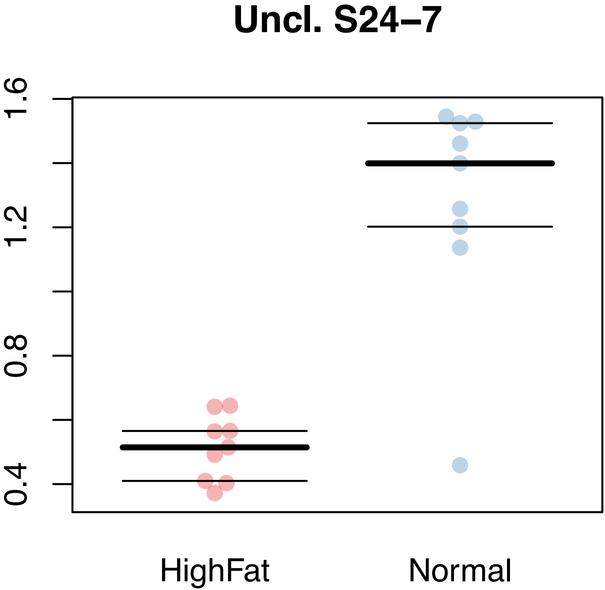
Uncl. S24–7
1.6
1.2
0.8
0.4
HighFat
Normal

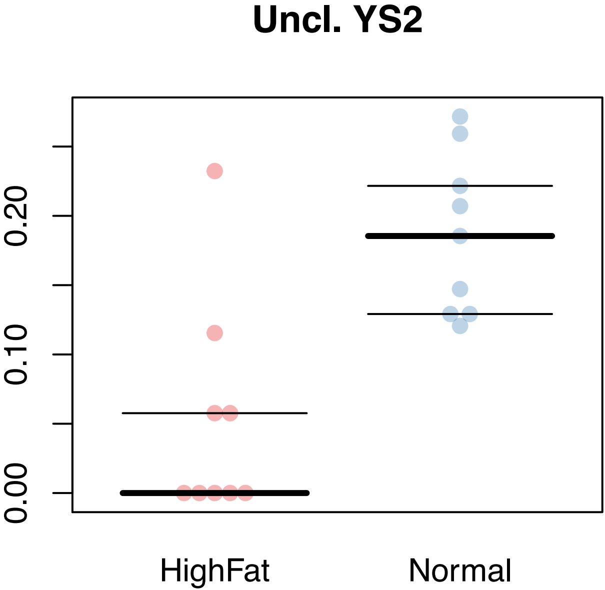

Uncl. YS2
0.00
0.10
0.20
HighFat
Normal

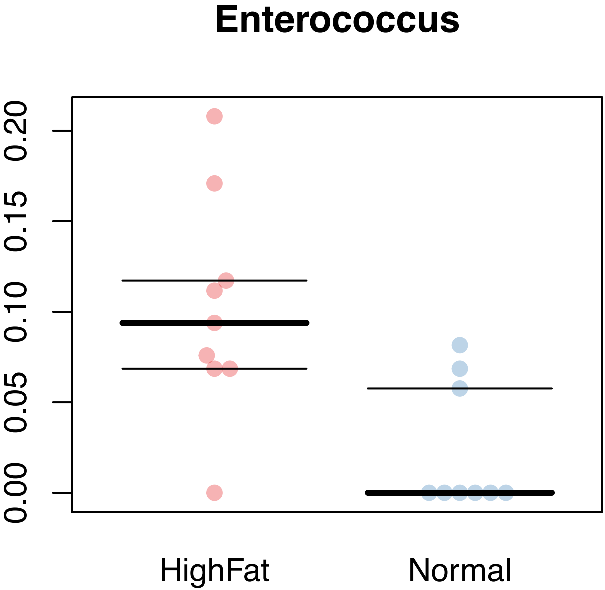
Enterococcus
0.00
0.05
0.10
0.15
0.20
HighFat
Normal

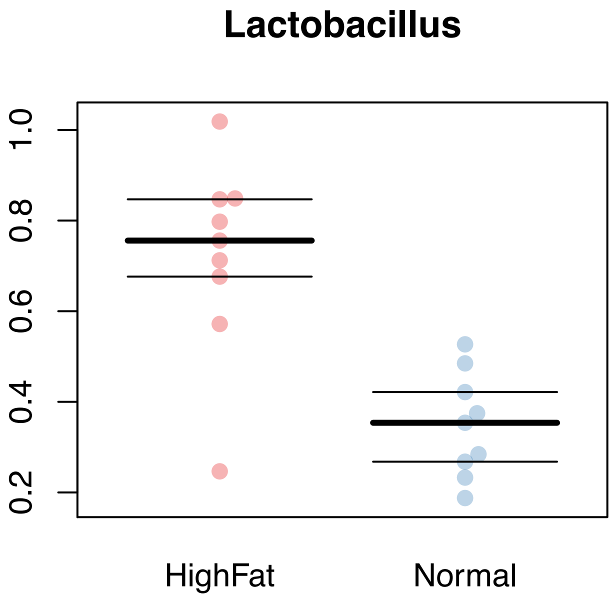
Lactobacillus
1.0
0.8
0.6
0.4
0.2
HighFat
Normal

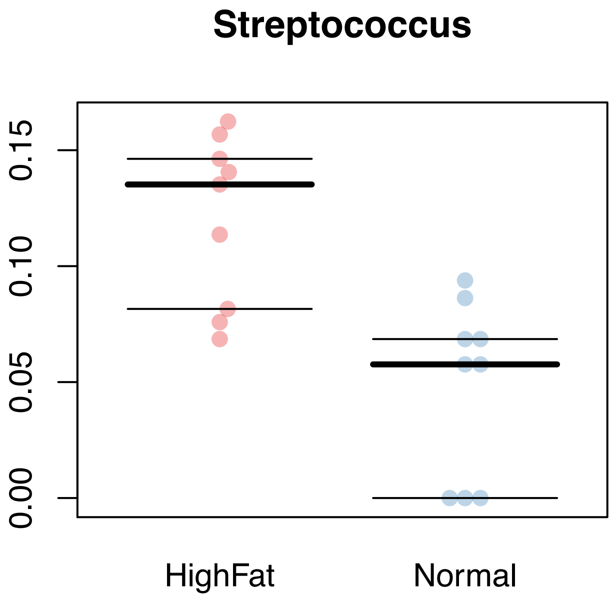
Streptococcus
0.00
0.05
0.10
0.15
HighFat
Normal

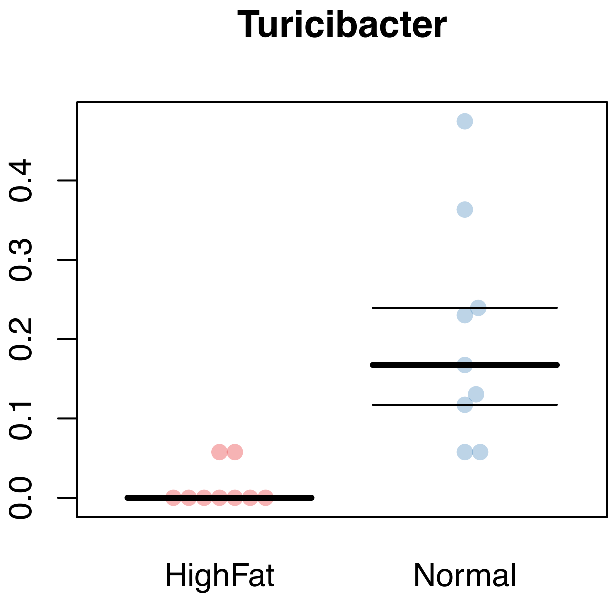
Turicibacter
0.0
0.1
0.2
0.3
0.4
HighFat
Normal

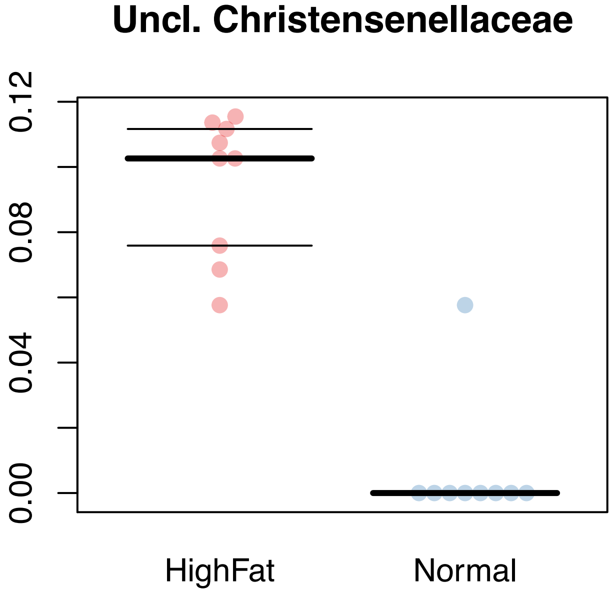
Uncl. Christensenellaceae
0.12
0.08
0.04
0.00
HighFat
Normal

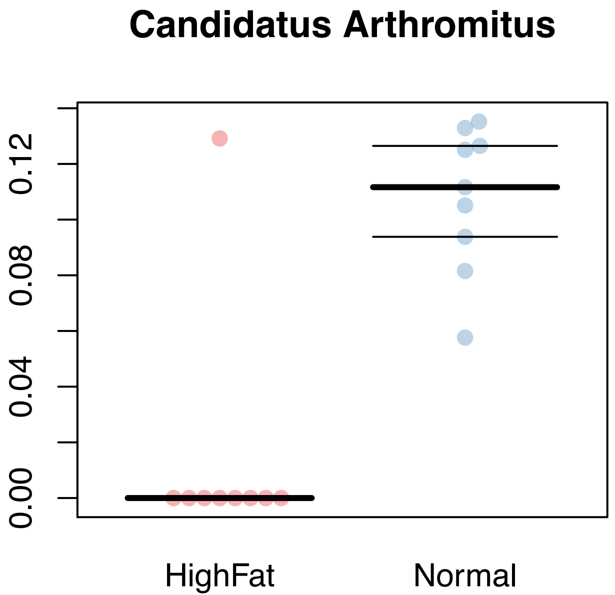

Candidatus Arthromitus
0.00
0.04
0.08
0.12
HighFat
Normal

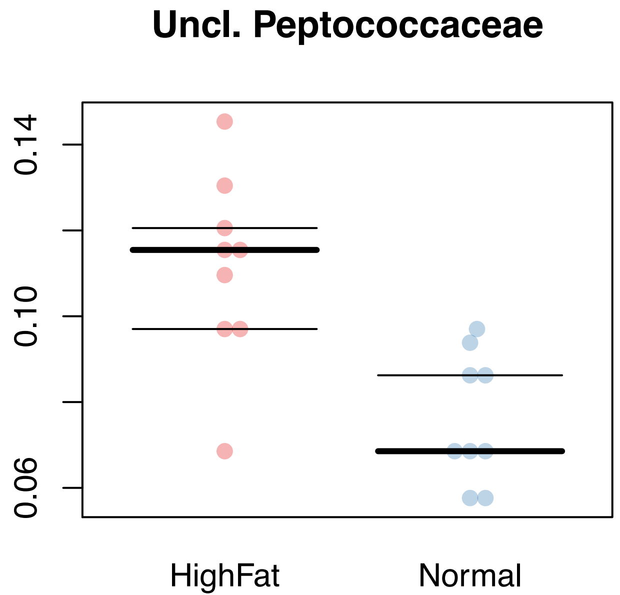
Uncl. Peptococcaceae
0.14
0.10
0.06
HighFat
Normal

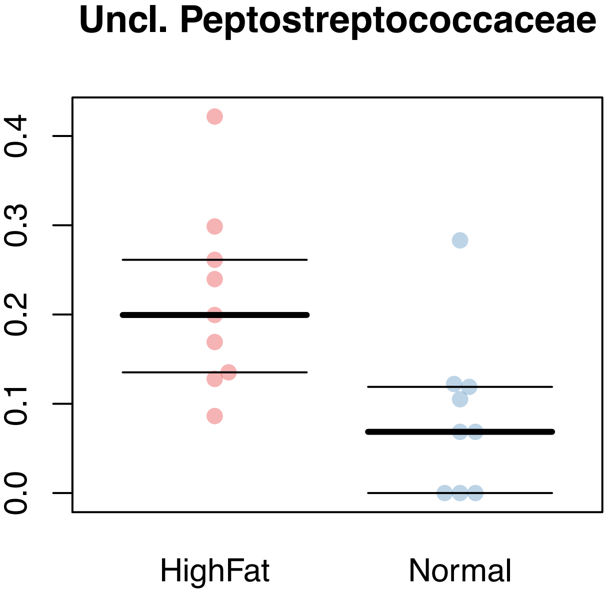

Uncl. Peptostreptococcaceae
0.0
0.1
0.2
0.3
0.4
HighFat
Normal

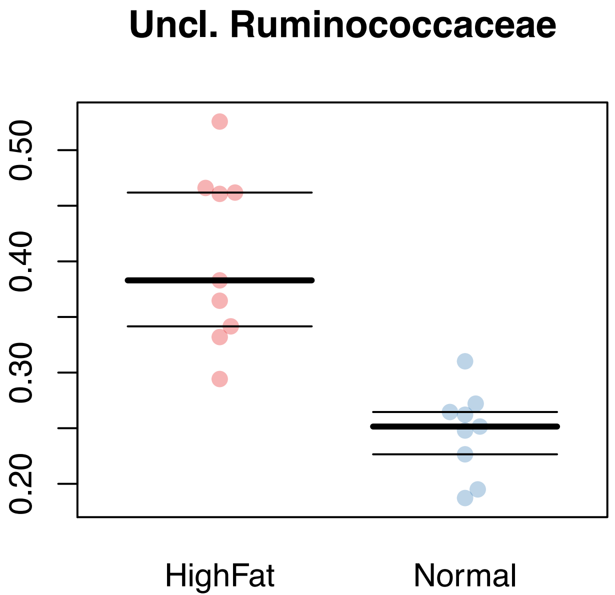
Uncl. Ruminococcaceae
0.20
0.30
0.40
0.50
HighFat
Normal

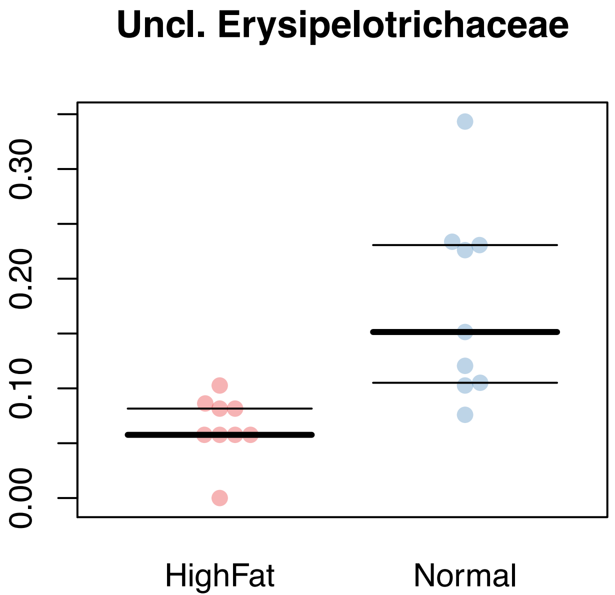

Uncl. Erysipelotrichaceae
0.00
0.10
0.20
0.30
HighFat
Normal

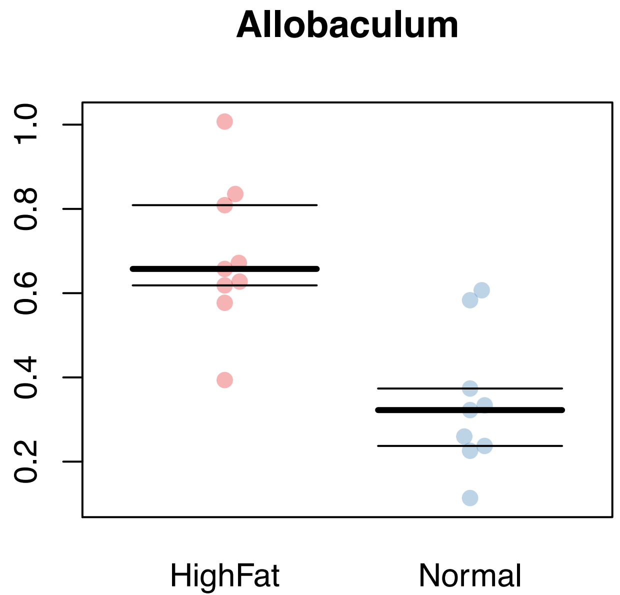

Allobaculum
1.0
0.8
0.6
0.4
0.2
HighFat
Normal

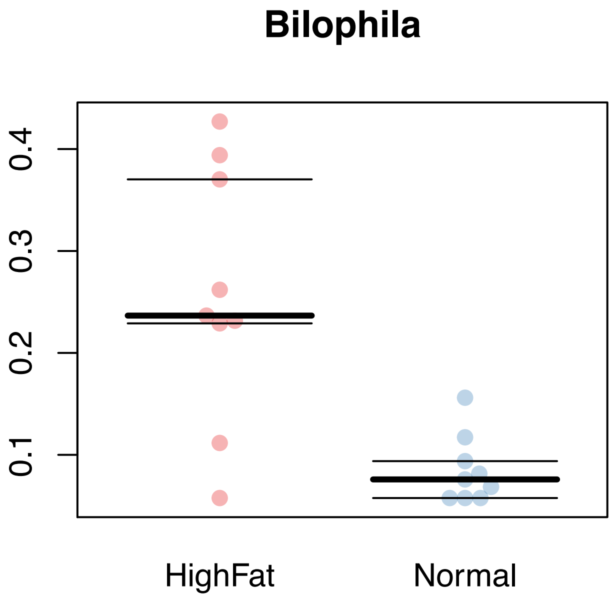
Bilophila
0.1
0.2
0.3
0.4
HighFat
Normal

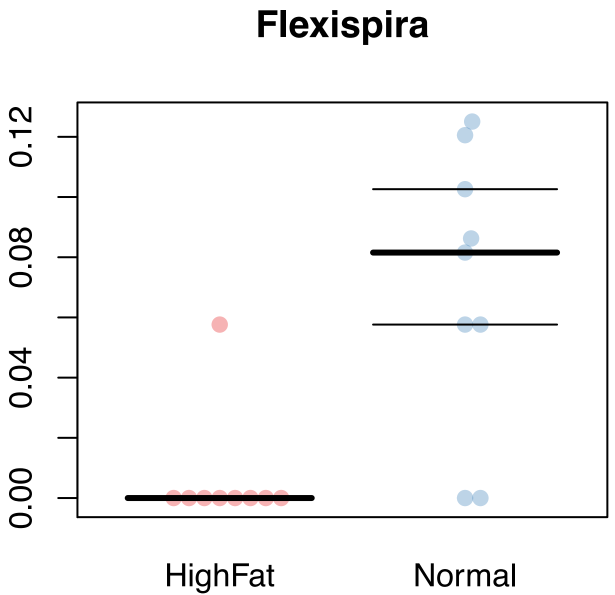
Flexispira
0.12
0.08
0.04
0.00
HighFat
Normal

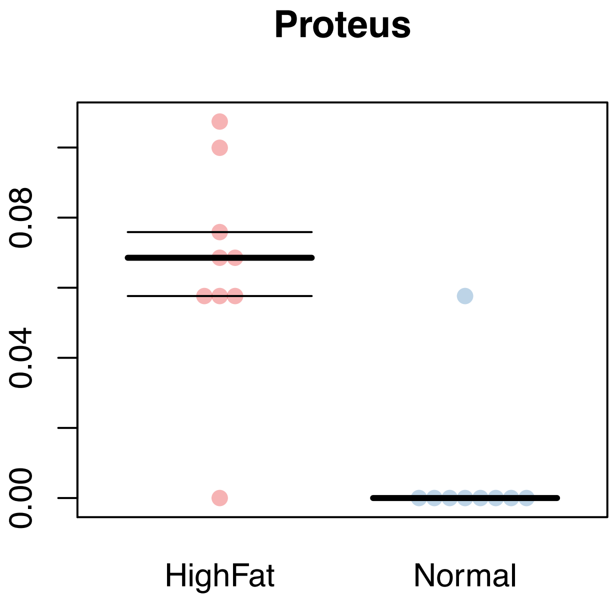
Proteus
0.00
0.04
0.08
HighFat
Normal

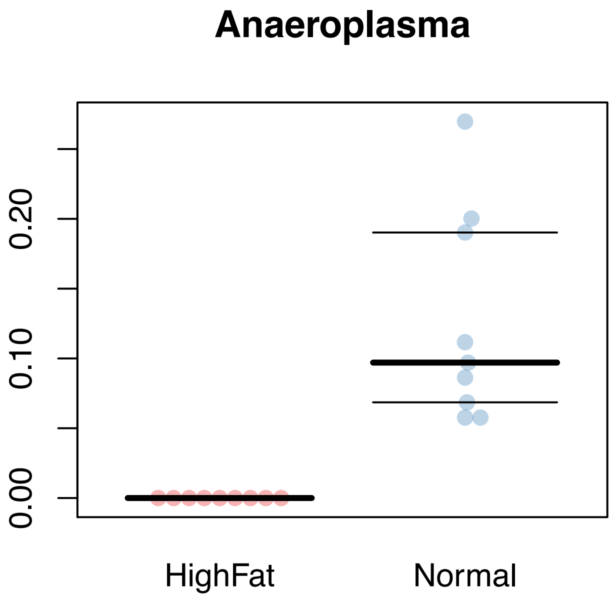
Anaeroplasma
0.00
0.10
0.20
HighFat
Normal
