# Supplementary material for: High-Fat Diet Changes Fungal Microbiomes and Interkingdom Relationships in the Murine Gut
Source: mSphere. 2017 Oct 11;2(5):e00351-17. doi: 10.1128/mSphere.00351-17 (PMC5636226; doi:10.1128/mSphere.00351-17)
Supplement: FIG S4 [file sph005172381sf4.pdf]

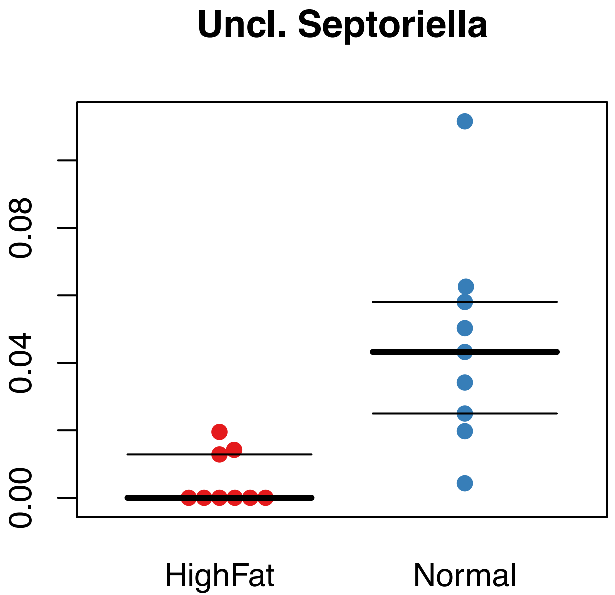

Uncl. Septoriella
0.00
0.04
0.08
HighFat
Normal

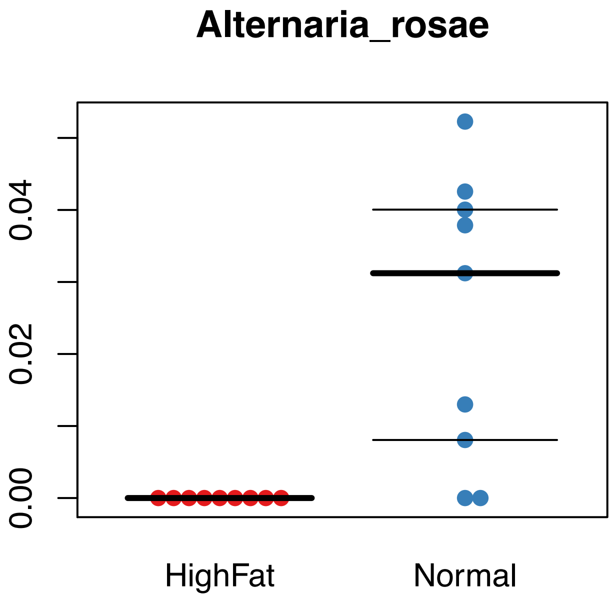
Alternaria_rosae
0.00
0.02
0.04
HighFat
Normal

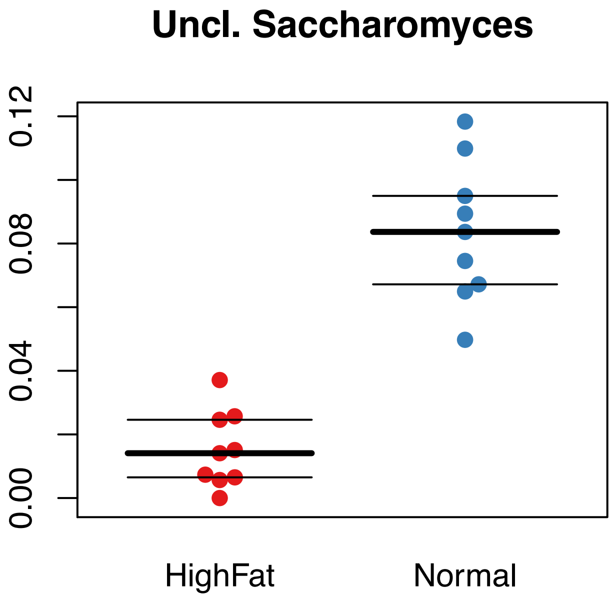
Uncl. Saccharomyces
0.00
0.04
0.08
0.12
HighFat
Normal

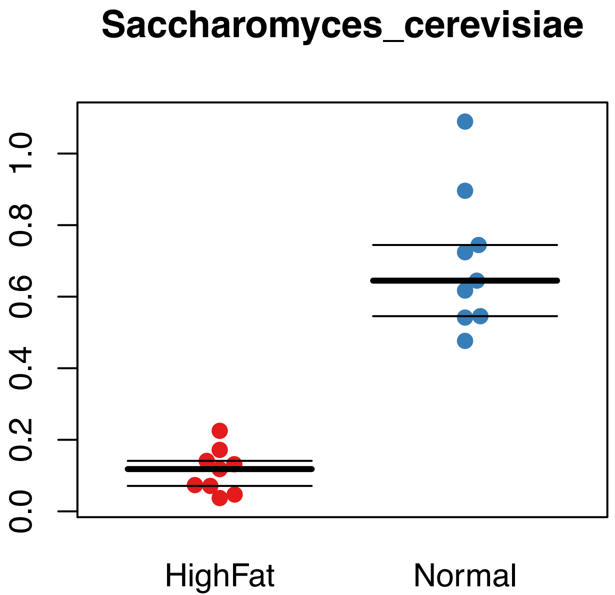

Saccharomyces_cerevisiae
0.0
0.2
0.4
0.6
0.8
1.0
HighFat
Normal

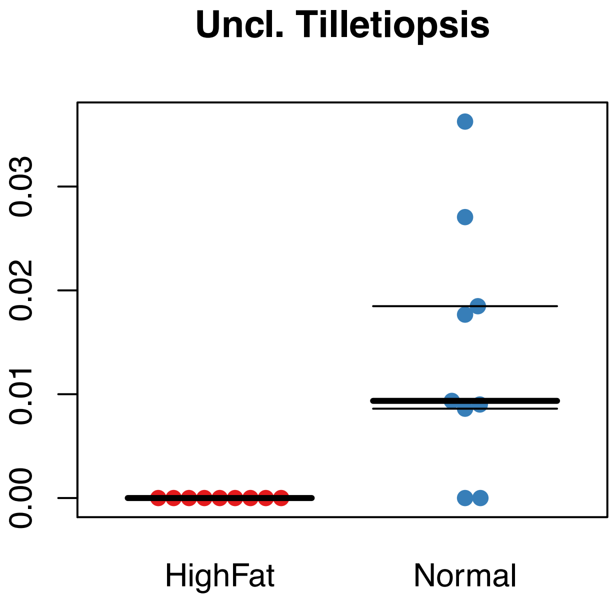

Uncl. Tilletiopsis
0.00
0.01
0.02
0.03
HighFat
Normal

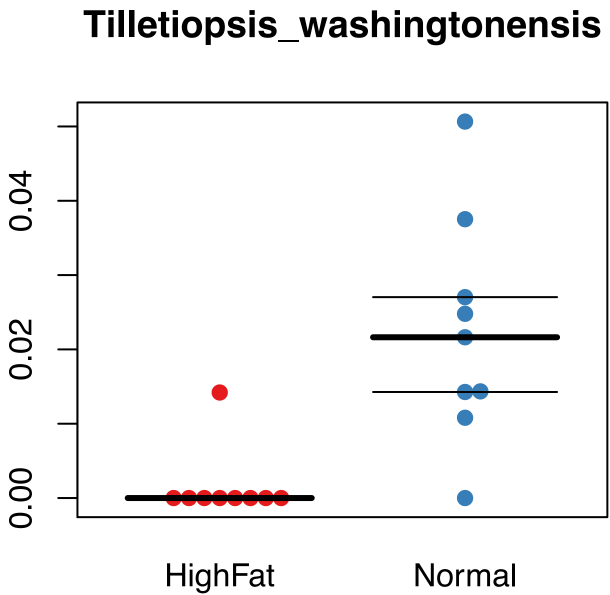

Tilletiopsis_washingtonensis
0.00
0.02
0.04
HighFat
Normal
