# Supplementary material for: High-Fat Diet Changes Fungal Microbiomes and Interkingdom Relationships in the Murine Gut
Source: mSphere. 2017 Oct 11;2(5):e00351-17. doi: 10.1128/mSphere.00351-17 (PMC5636226; doi:10.1128/mSphere.00351-17)
Supplement: FIG S5 [file sph005172381sf5.pdf]

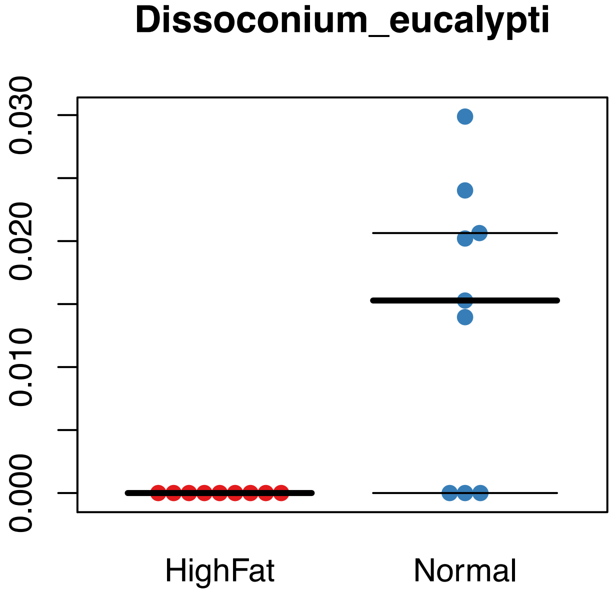
Dissoconium_eucalypti
0.000
0.010
0.020
0.030
HighFat
Normal

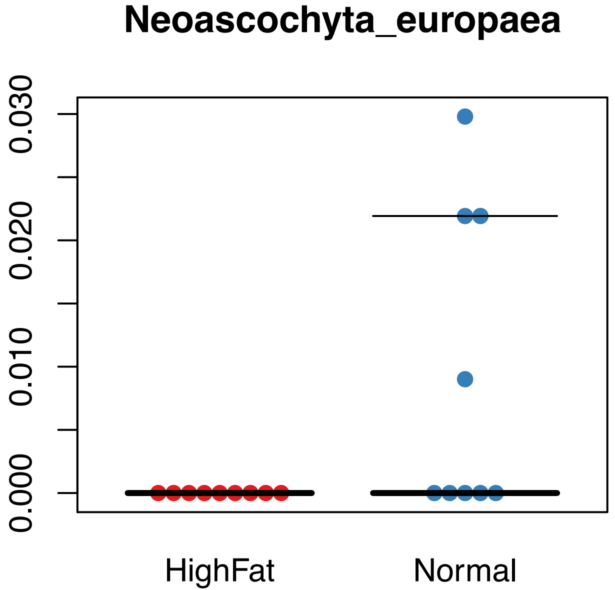
Neoascochyta_europaea
0.000
0.010
0.020
0.030
HighFat
Normal

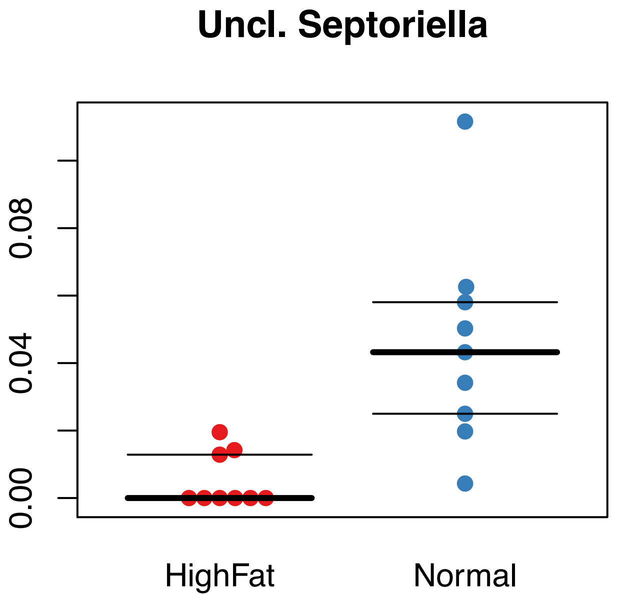

Uncl. Septoriella
0.00
0.04
0.08
HighFat
Normal

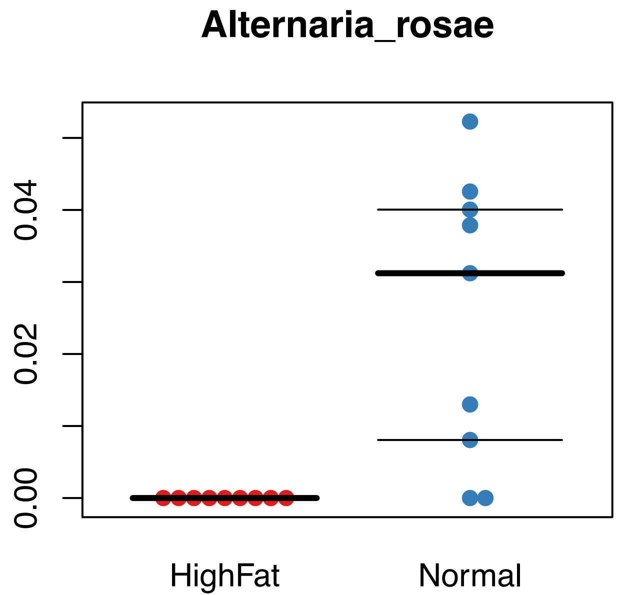
Alternaria_rosae
0.00
0.02
0.04
HighFat
Normal

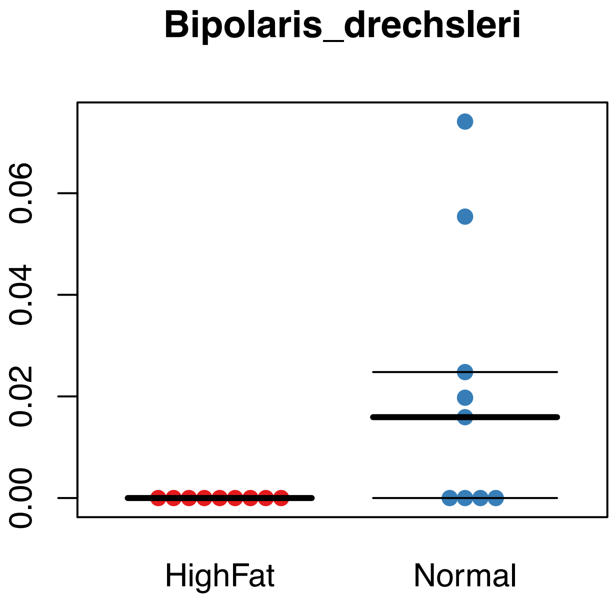
Bipolaris_drechsleri
0.00
0.02
0.04
0.06
HighFat
Normal

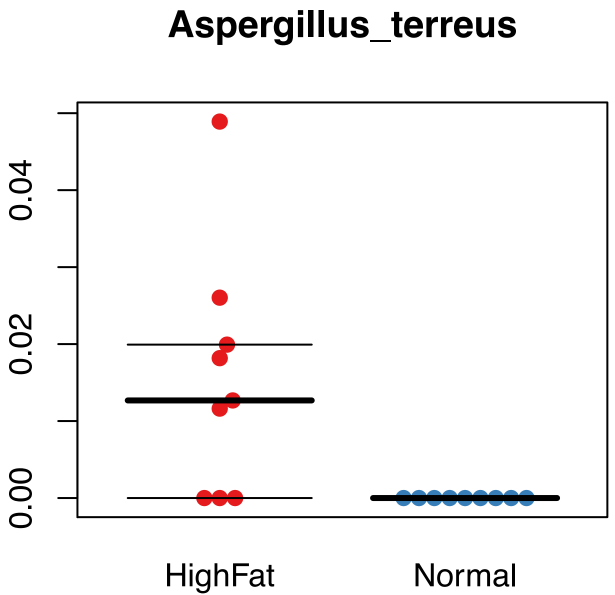
Aspergillus_terreus
0.00
0.02
0.04
HighFat
Normal

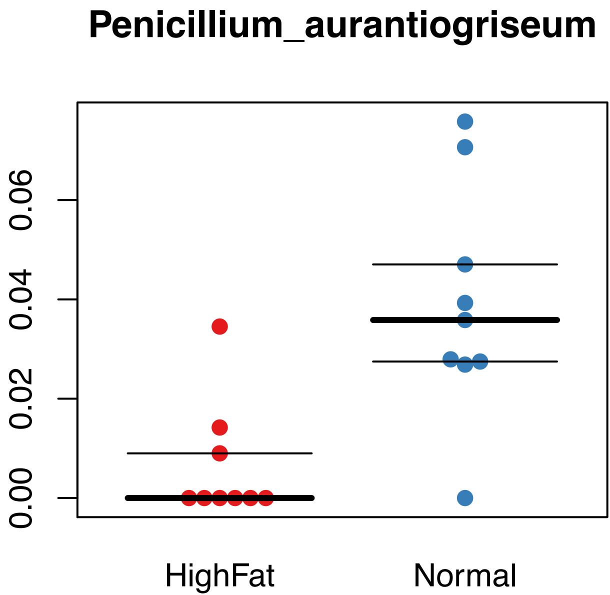
Penicillium_aurantiogriseum
0.00
0.02
0.04
0.06
HighFat
Normal

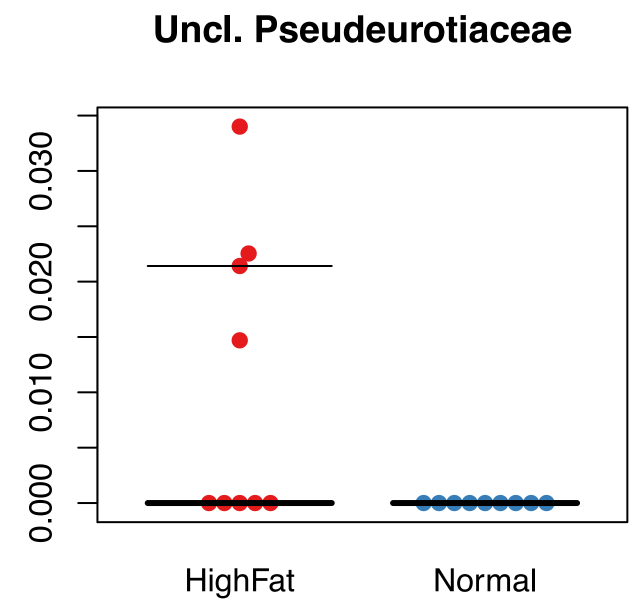

Uncl. Pseudeurotiaceae
0.030
0.020
0.010
0.000
HighFat
Normal

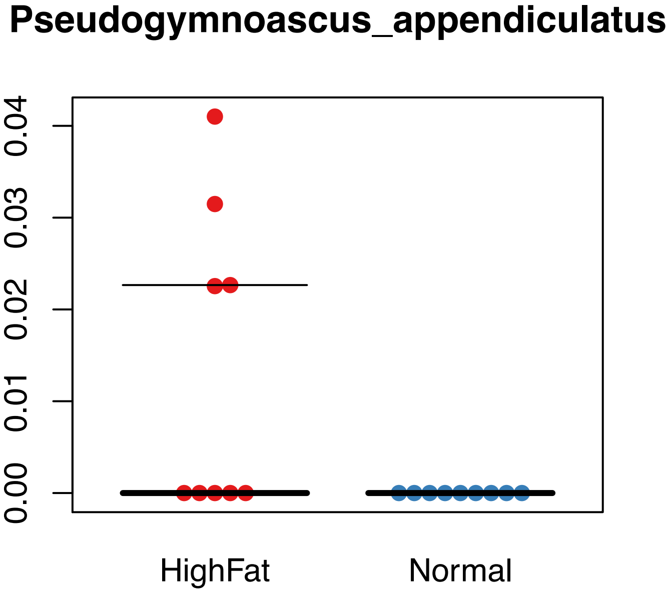
Pseudogymnoascus_appendiculatus
0.04
0.03
0.02
0.01
0.00
HighFat
Normal

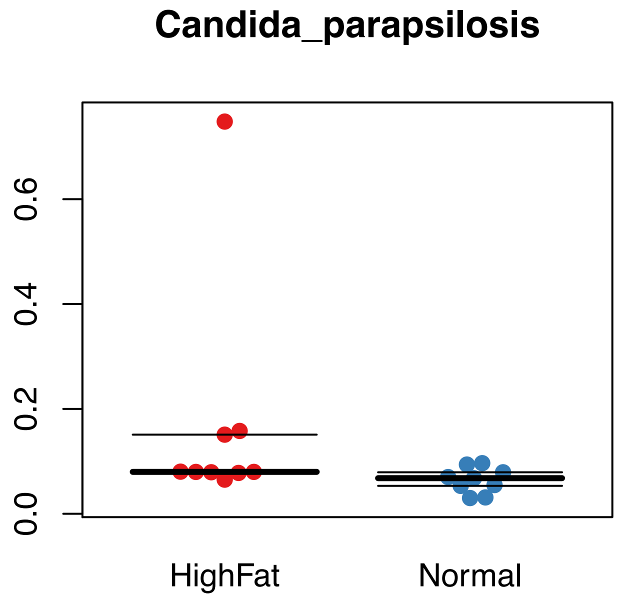
Candida_parapsilosis
0.0
0.2
0.4
0.6
HighFat
Normal

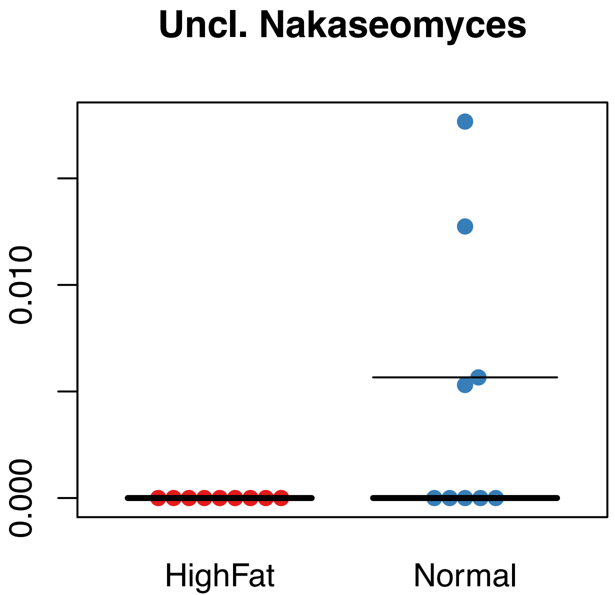
Uncl. Nakaseomyces
0.000
0.010
HighFat
Normal

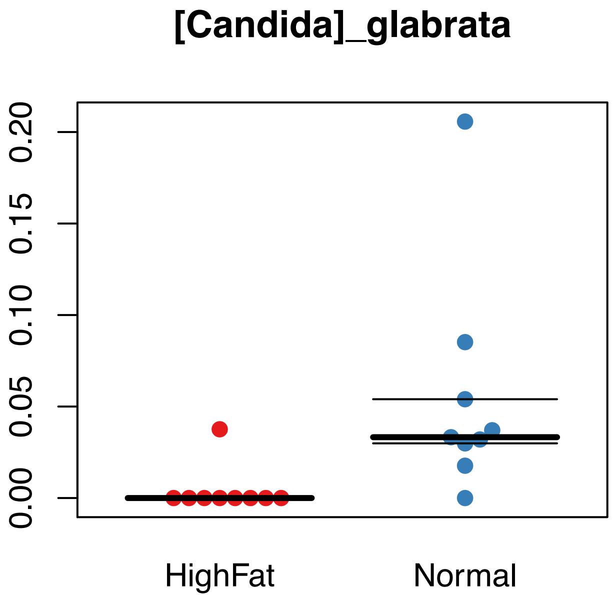

[Candida]_glabrata
0.00
0.05
0.10
0.15
0.20
HighFat
Normal

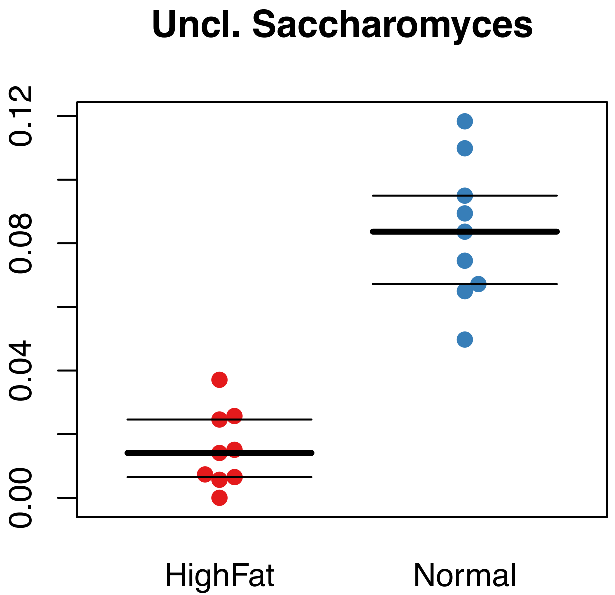

Uncl. Saccharomyces
0.00
0.04
0.08
0.12
HighFat
Normal

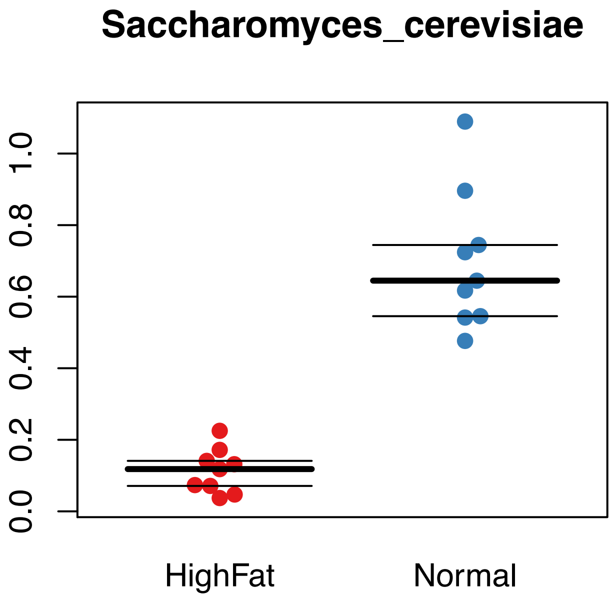
Saccharomyces_cerevisiae
0.0
0.2
0.4
0.6
0.8
1.0
HighFat
Normal

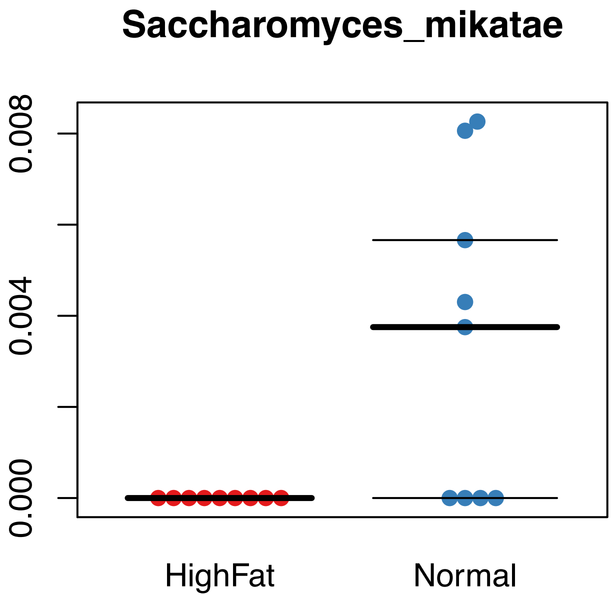

Saccharomyces_mikatae
0.000
0.004
0.008
HighFat
Normal

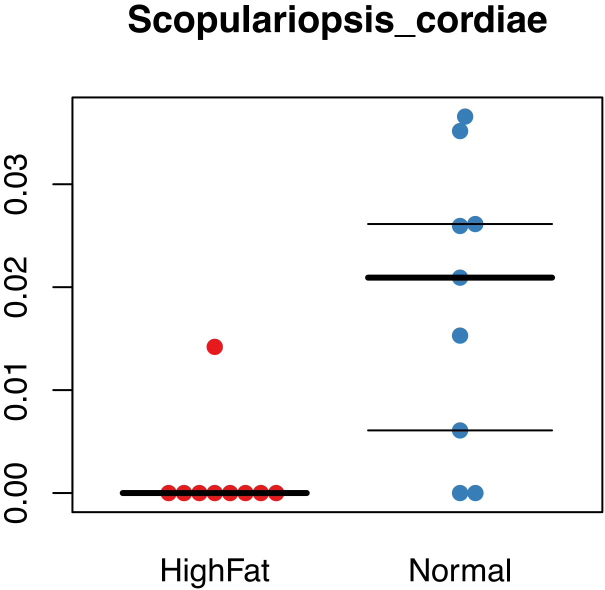
Scopulariopsis_cordiae
0.00
0.01
0.02
0.03
HighFat
Normal

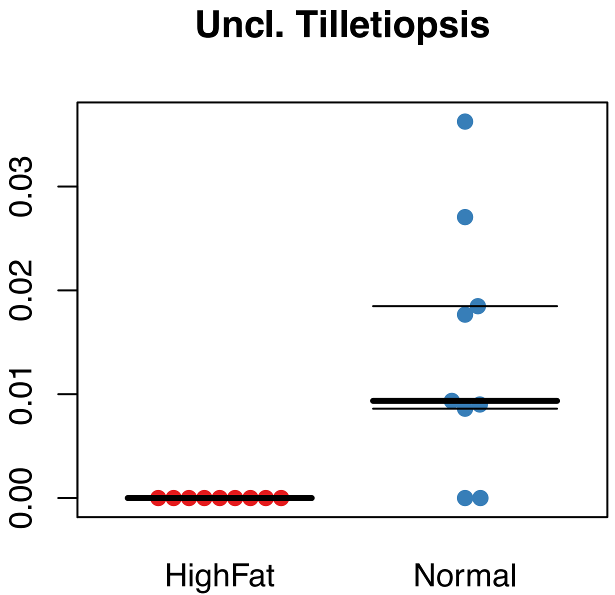
Uncl. Tilletiopsis
0.00
0.01
0.02
0.03
HighFat
Normal

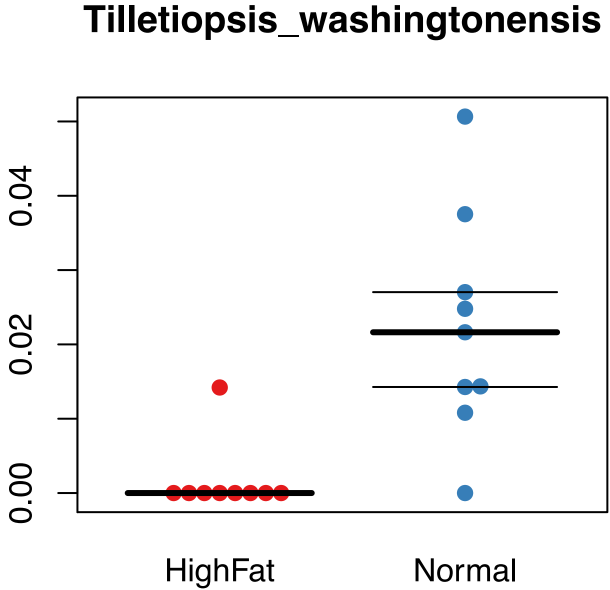
Tilletiopsis_washingtonensis
0.00
0.02
0.04
HighFat
Normal

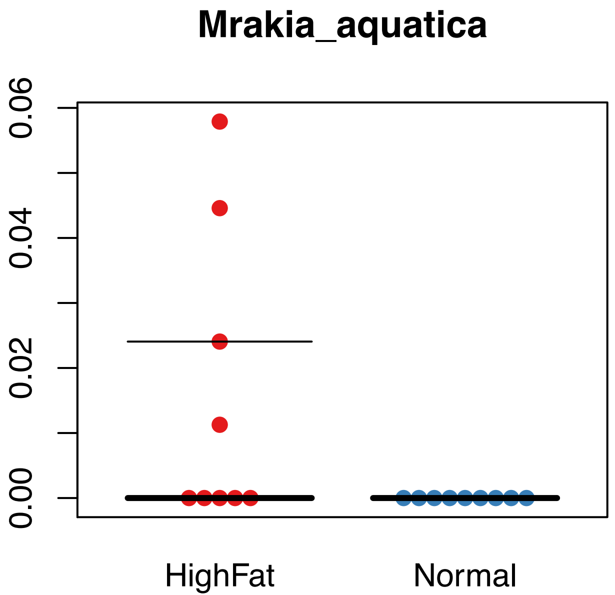
Mrakia_aquatica
0.06
0.04
0.02
0.00
HighFat
Normal

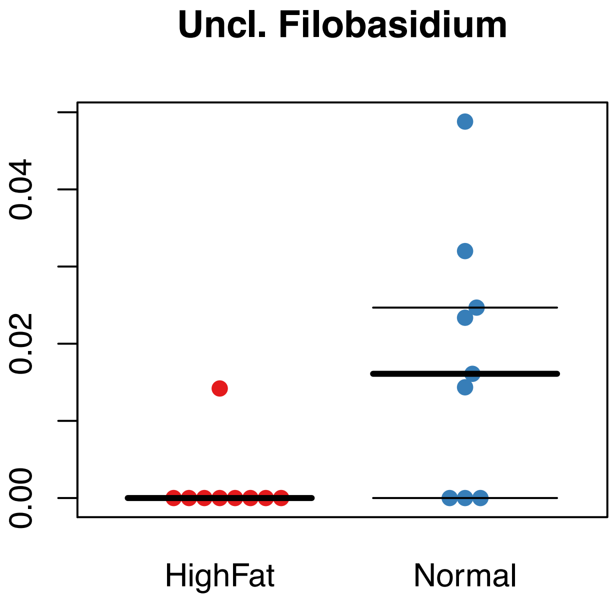
Uncl. Filobasidium
0.00
0.02
0.04
HighFat
Normal

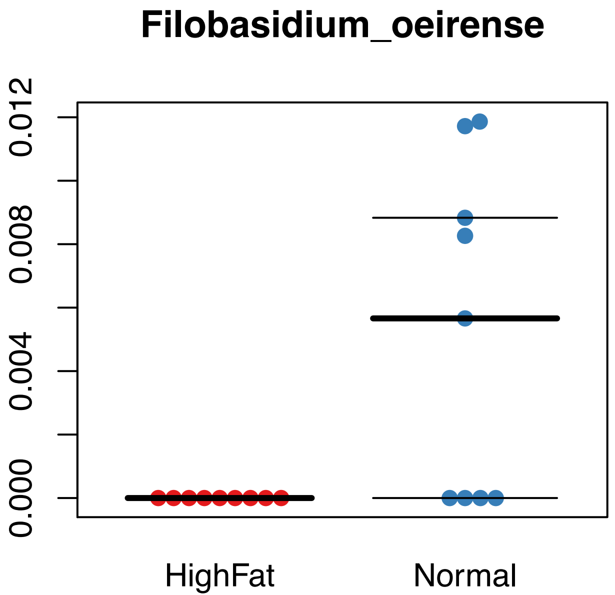

Filobasidium_oeirense
0.000
0.004
0.008
0.012
HighFat
Normal

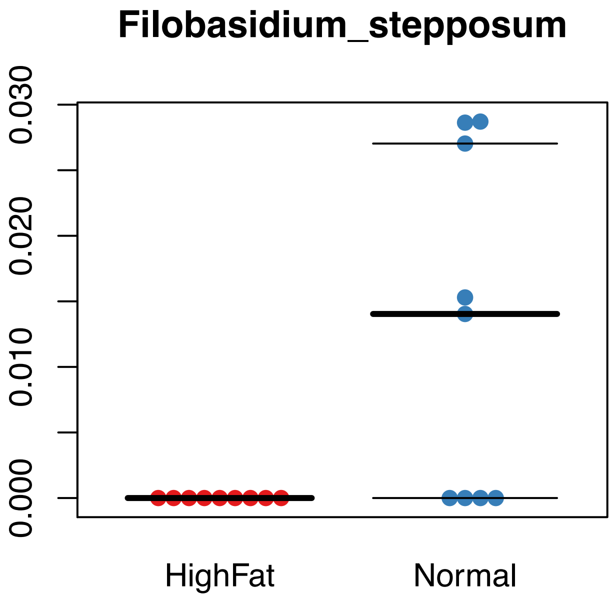
Filobasidium_stepposum
0.000
0.010
0.020
0.030
HighFat
Normal

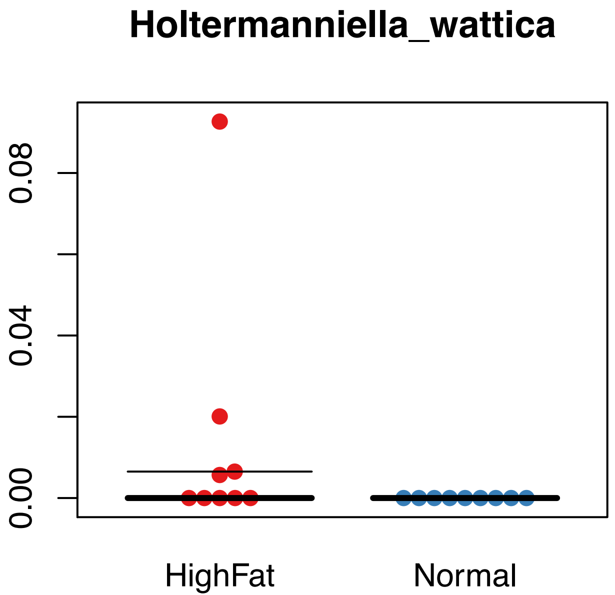

Holtermanniella_wattica
0.00
0.04
0.08
HighFat
Normal

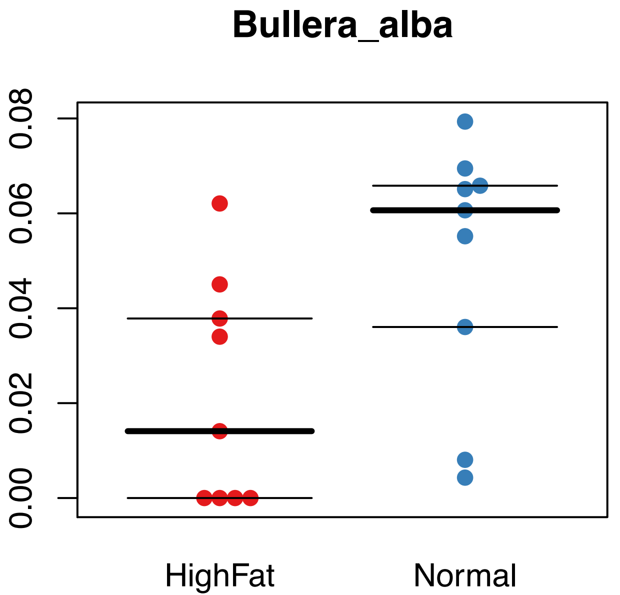

Bullera_alba
0.00
0.02
0.04
0.06
0.08
HighFat
Normal

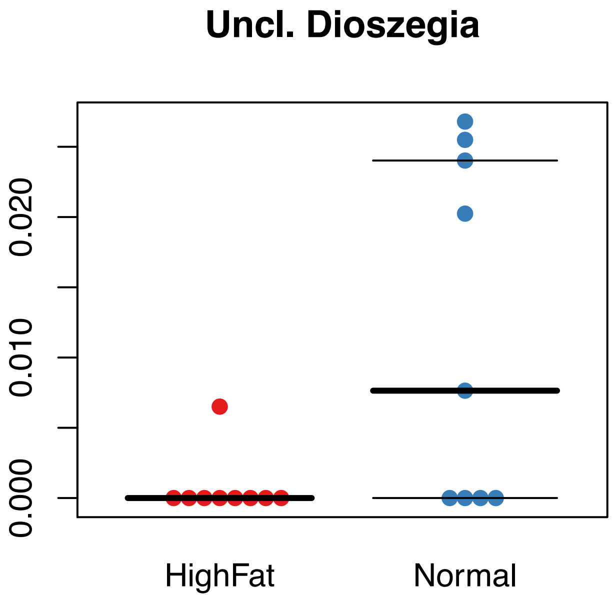
Uncl. Dioszegia
0.000
0.010
0.020
HighFat
Normal

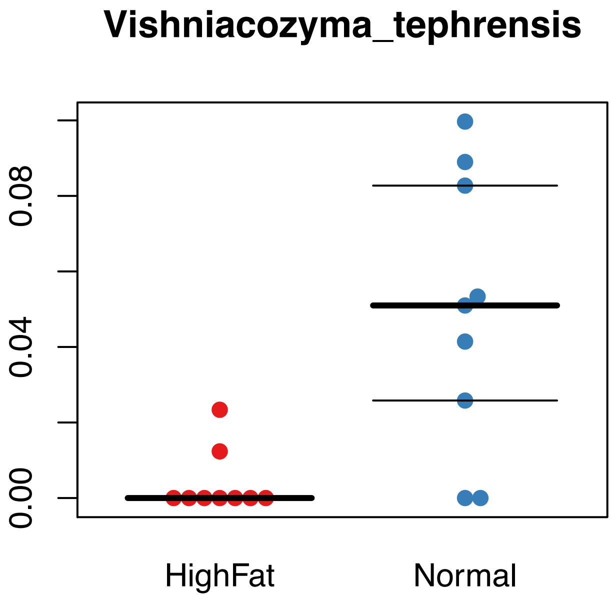
Vishniacozyma_tephrensis
0.00
0.04
0.08
HighFat
Normal

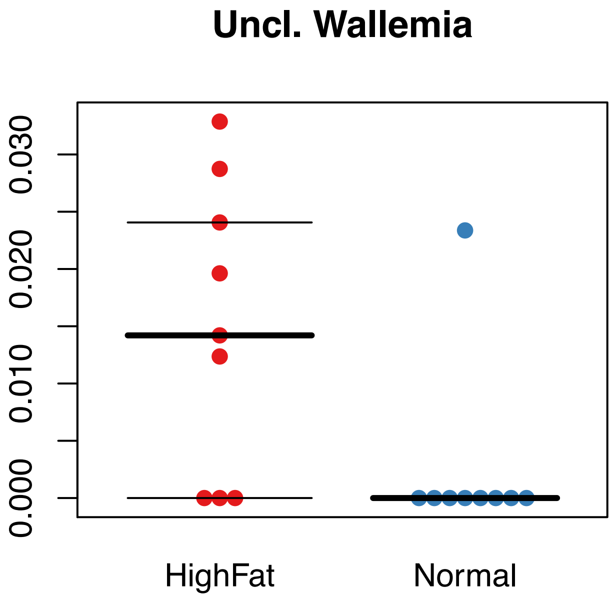
Uncl. Wallemia
0.000
0.010
0.020
0.030
HighFat
Normal
